# Association of Bipolar Disorder Diagnosis With Suicide Mortality Rates in Adolescents in Sweden

**DOI:** 10.1001/jamapsychiatry.2023.1390

**Published:** 2023-05-24

**Authors:** Peter Andersson, Jussi Jokinen, Håkan Jarbin, Johan Lundberg, Adrian E. Desai Boström

**Affiliations:** 1 Department of Clinical Neuroscience/Psychology, Karolinska Institutet, Stockholm, Sweden; 2 Centre for Clinical Research Dalarna, Uppsala University, Uppsala, Sweden; 3 Centre for Psychiatry Research, Department of Clinical Neuroscience, Karolinska Institutet, and Stockholm Health Care Services, Region Stockholm, Karolinska University Hospital, Stockholm, Sweden; 4 Department of Clinical Sciences/Psychiatry, Umeå University, Umeå, Sweden; 5 Section of Child and Adolescent Psychiatry, Department of Clinical Sciences, Lund University, Lund, Sweden; 6 Child and Adolescent Psychiatry, Region Halland, Halmstad, Sweden; 7 Stockholm Health Care Services, Region Stockholm, Stockholm, Sweden

## Abstract

**Importance:**

The association of early diagnosis and management of bipolar disorder with adolescent suicide mortality (ASM) is unknown.

**Objective:**

To assess regional associations between ASM and bipolar disorder diagnosis frequencies.

**Design, Setting, and Participants:**

This cross-sectional study investigated the association between annual regional ASM and bipolar disorder diagnosis rates in Swedish adolescents aged 15 to 19 years in January 1, 2008, through December 31, 2021. Aggregated data without exclusions reported at the regional level encompassed 585 suicide deaths, constituting 588 unique observations (ie, 21 regions, 14 years, 2 sexes).

**Exposures:**

Bipolar disorder diagnosis frequencies and lithium dispensation rates were designated as fixed-effects variables (interaction term in the case of males). An interaction term between psychiatric care affiliation rates and the proportion of psychiatric visits to inpatient and outpatient clinics constituted independent fixed-effects variables. Region and year comprised random intercept effect modifiers. Variables were population adjusted and corrected for heterogeneity in reporting standards.

**Main Outcomes and Measures:**

The main outcomes were sex-stratified, regional, and annual ASM rates in adolescents aged 15 to 19 years per 100 000 inhabitants as analyzed using generalized linear mixed-effects models.

**Results:**

Female adolescents were diagnosed with bipolar disorder almost 3 times more often than male adolescents (mean [SD], 149.0 [19.6] vs 55.3 [6.1] per 100 000 inhabitants, respectively). Median regional prevalence rates of bipolar disorder varied over the national median by a factor of 0.46 to 2.61 and 0.00 to 1.82 in females and males, respectively. Bipolar disorder diagnosis rates were inversely associated with male ASM (β = −0.00429; SE, 0.002; 95% CI, −0.0081 to −0.0004; *P* = .03) independent of lithium treatment and psychiatric care affiliation rates. This association was replicated by β-binomial models of a dichotomized quartile 4 ASM variable (odds ratio, 0.630; 95% CI, 0.457-0.869; *P* = .005), and both models were robust after adjusting for annual regional diagnosis rates of major depressive disorder and schizophrenia. No such association was observed in females.

**Conclusions and Relevance:**

In this cross-sectional study, lower suicide death rates in adolescent males was robustly associated with regional diagnosis rates of bipolar disorder at an estimated magnitude of approximately 4.7% of the mean national suicide death rate. The associations could be due to treatment efficacy, early diagnosis and management, or other factors not accounted for.

## Key Points

**Question:**

Is diagnosing bipolar spectrum disorder in adolescents associated with suicide prevention?

**Findings:**

In this cross-sectional study of 585 confirmed suicide deaths in Sweden, regional bipolar disorder diagnosis rates in adolescent males were associated with a lower suicide death rate at an estimated magnitude of approximately 4.7% of the national average. Independent of annual regional depression and schizophrenia diagnoses, lithium dispensation, and psychiatric care affiliation rates, results were consistent with previous reports suggesting that bipolar disorder is implicated in approximately 4.9% of unselected suicide deaths in young adulthood.

**Meaning:**

The findings indicate that diagnosis of bipolar disorder in male adolescents may be important for suicide prevention.

## Teaser

This cross-sectional study uses national Swedish registry data to assess regional associations of bipolar disorder diagnosis rates and suicide mortality in adolescents.

## Introduction

Bipolar spectrum disorder is a chronic severe mental illness characterized by recurring episodes of elevated mood and depression with a general peak age of onset at 12 to 25 years.^1,2^ Long delays have been observed from disease onset to diagnosis and initiation of treatment. A recently published meta-analysis found median times from onset to help seeking, diagnosis, and initiation of mood-stabilizing pharmacotherapy of 3.5, 6.7, and 5.9 years, respectively.^3^ Factors influencing latencies were sex, treatment setting, aspects of disease presentation, and geographic location, with shorter delays to diagnosis observed in Europe and Scandinavia vs North America.^3^ The diagnosis of bipolar disorder in pediatric populations is a widely discussed topic in the literature, particularly due to conflicting perceptions of prevalence and treatment, as well as the potential role of irritability.^4^ The National Comorbidity Survey Replication has estimated that bipolar disorder affects 2.8% of US adults and 2.9% of US adolescents, with higher prevalence among adolescent females (3.3%) vs adolescent males (2.6%), but largely equal prevalence rates across the sexes in adults.^5,6^ Van Meter et al^7^ reported a weighted average prevalence of youth bipolar disorder to be 3.9% (95% CI, 2.6%-5.8%). The authors suggested that heterogenous prevalence rates could be attributed to nonstandard diagnostic criteria and narrow definitions. Their study also indicated that prevalence rates in the US are not higher than in other Western countries and that there is no increasing trend over time. Moreover, in a review of the evidence base, Findling et al^4^ found consensus favoring diagnosis according to *DSM-5* criteria, consistent with the report by Perlis et al,^8^ that a majority of adult patients with bipolar disorder presented with onset of mood symptoms in youth (aged <18 years) and that early onset was associated with more recurrences, greater rates of substance abuse, and a greater likelihood of suicide attempts and violence. Emerging evidence supports psychological and pharmacologic treatments in early disease stages to improve outcomes.^9,10^ Nevertheless, practices of diagnosing bipolar disorder in youth have been controversial, and issues related to a perceived lack of awareness, diagnostic confusion, stigma, and other factors may have negatively affected diagnosis rates.^11^ Accordingly, international clinical guidelines communicate incongruent recommendations for the diagnosis of youth bipolar disorder. For example, National Institute for Health and Care Excellence guidelines posit substantially stricter criteria for diagnosing bipolar disorder in adolescents vs adults (eg, requiring the presence of mania),^12^ whereas the American Academy of Child and Adolescent Psychiatry recommends adhering to traditional *DSM* criteria.^13^

Epidemiologic reviews have suggested that 44% to 76% of adolescents who die by suicide meet criteria for an affective disorder.^14^ The exact contribution of bipolar disorder to suicide deaths in adolescence is not fully elucidated. Clements et al,^15^ however, reviewed registered suicides in a national English sample during 1996 to 2009, and found that 4.9% (57 of 1163) of suicides in young adulthood (aged ≤24 years) had a primary diagnosis of bipolar disorder. Lithium treatment has shown greater efficacy in reducing suicide attempts in youth with bipolar disorder compared with other mood-stabilizing treatments.^16^ However, the exact ratio of attempted suicides to suicide deaths in adolescents with bipolar disorder remains unclear and is estimated to be between 50:1 and 100:1 in the general adolescent population.^17^ This uncertainty complicates the interpretation of the potential suicide-protective effects of lithium in this specific population.

The aim of our study was 2-fold. First, we hypothesized that the historical lack of solid evidence in support of practices for diagnosing bipolar disorder in youth may have contributed to health inequities regarding diagnosis and treatment rates at the regional level. Our goal was to comprehensively examine and compare the national prevalence of bipolar disorder in its various forms (bipolar 1, 2, and not otherwise specified) across the 21 regions of Sweden during the years 2008 to 2021 in individuals aged 15 to 19 years. Additionally, we aimed to investigate the potential association between bipolar disorder diagnosis frequencies and the total regional dispensation of lithium treatment within this age group. Second, we hypothesized that regional diagnosis rates of bipolar disorder and treatment with lithium are associated with reduced suicide death rates in adolescents, ie, that early diagnosis (and management) of bipolar disorder and/or lithium treatment would bestow reduced adolescent suicide mortality (ASM) at the regional level.

## Methods

### Study Design and Patients

In this cross-sectional study of sex-stratified suicide death rates, bipolar disorder diagnosis frequencies, and lithium dispensation rates in youth aged 15 to 19 years, data were retrieved for the 21 Swedish regions from January 1, 2008, through December 31, 2021, from the Swedish National Board of Health and Welfare,^18^ a freely available Swedish data set.^19,20,21^ Extracted data included registered bipolar disorder diagnosis frequencies (*ICD-10* code F31) in both specialized outpatient and inpatient care and confirmed suicide death rates (codes X60-X84) per 100 000 inhabitants, as well as the number of dispensations to adolescents recorded for lithium (Anatomical Therapeutic Chemical code N05AN01) per 1000 inhabitants in each region and age group, respectively. This work was conducted in accordance with the ethical standards of the Declaration of Helsinki and in accordance with Swedish laws on research ethics. As the study pertained to openly available data, no ethical permission or informed consent for publication were required by Swedish jurisdiction. Karolinska Institutet regulatory standards were, however, followed. The study followed the Strengthening the Reporting of Observational Studies in Epidemiology (STROBE) reporting guideline.^22^ Sources for additional control variables and initial processing steps are detailed in the eMethods in Supplement 1.

The full sample encompassed aggregated data at the regional level, representing all registered Swedish citizens who were aged 15 to 19 years between 2008 and 2021, died by suicide, and/or were diagnosed with bipolar disorder (stratified by region and sex). The sample included 585 confirmed suicide deaths and 8033 cases of bipolar disorder. The aggregated data used for subsequent analyses included 588 unique observations, or 294 observations for each sex and variable (ie, 21 regions, 14 years, 2 sexes). No data were excluded from downstream analyses.

### Statistical Analysis

Prior to statistical association analyses, several measures were taken to strengthen robustness and reduce bias from putative sources of confounding. Details are provided in the eMethods in Supplement 1.

#### Main Models

Associations between annual bipolar disorder diagnosis frequencies and lithium dispensation rates were investigated using generalized linear mixed-effects (GLME) models, whereby the outcome variable (regional bipolar disorder diagnosis frequencies) was contrasted with fixed-effects exposure variables (ie, lithium dispensation rates and an interaction term between the reporting standard–adjusted outpatient psychiatric care affiliation rates [PCAR] and reporting standard–adjusted proportion of inpatient to outpatient visits [OutInQuota], factors that could be associated with regional variations in suicide rates^23,24^) and random intercept effect modifiers (region and year). Based on previous research implicating sex differences in suicide-related outcomes^14^ and in the presentation of pediatric bipolar disorder,^25^ males and females were studied separately.

To avoid violating model assumptions, PCAR and OutInQuota were subjected to normalization by the Blom method^26^ for all models, maintaining the mutual relationship between observations. Associations between bipolar disorder diagnosis rates and lithium dispensation frequencies were found in males but not females. To reduce confounding from collinearity, downstream analyses accounted for an interaction term between bipolar disorder and lithium rates in the case of males but treated these variables as independent from each other in females. Associations between the outcome variable (regional suicide death rates) and exposures (bipolar disorder diagnosis frequencies, lithium dispensation rates, PCAR, and OutInQuota) were then investigated separately for each sex by GLME models, whereby regional bipolar disorder diagnosis frequencies and annual dispensations for lithium treatment were designated as fixed-effects variables (interaction term in the case of males) and a fixed-effects interaction term between PCAR and OutInQuota, and random intercept effect modifiers (region and year) were treated as random effects. In this analysis, the following variables were subjected to normalization by the Blom method^26^ to avoid violating model assumptions: lithium dispensation rates, PCAR, and OutInQuota. Additional post hoc analyses to ensure the robustness and validity of the results obtained from the analyses are detailed in the eMethods in Supplement 1.

#### Validation Models

Significant models were further explored by separate analyses. The annual regional ASM variable was dichotomized based on the 75th percentile, whereby ASM observations in quartile 4 were compared with quartile 1 to quartile 3 observations. To account for the binomial distribution of the dichotomized response variable, GLME models modeled on the β-binomial distribution were implemented. Similarly, 2 independent interaction terms were specified as fixed effects (1 between regional bipolar disorder diagnosis frequencies and annual dispensations for lithium treatment and 1 between PCAR and OutInQuota), and region and year were treated as random effects. All exposure variables were normalized using the Blom method^26^ to avoid violating model assumptions. A 2-sided *P* < .05 was considered significant. All statistical analyses were performed in using R, version 4.0.3 software (R Foundation for Statistical Computing).

## Results

### Baseline Characteristics of Data

**Figure 1.**
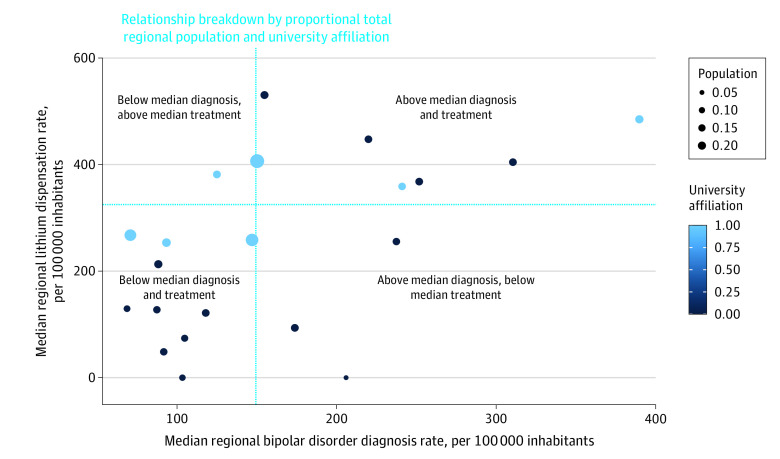
Median Regional Frequencies of Bipolar Disorder Diagnosis and Lithium Treatment in Female Adolescents Aged 15 to 19 Years, 2008-2021 Straight lines illustrate the national median for the same age group and period. University-affiliated counties are highlighted in blue, others in black. County population, retrieved from the Swedish Central Bureau of Statistics, is illustrated by circle diameter. There were large regional variations in bipolar disorder diagnosis frequencies and lithium dispensation rates in adolescent females. Median regional prevalence and prescription rates of bipolar disorder and lithium deviated over the national median by a factor of 0.46 to 2.61 for bipolar disorder diagnosis and 0.00 to 1.75 for lithium treatment.

**Figure 2.**
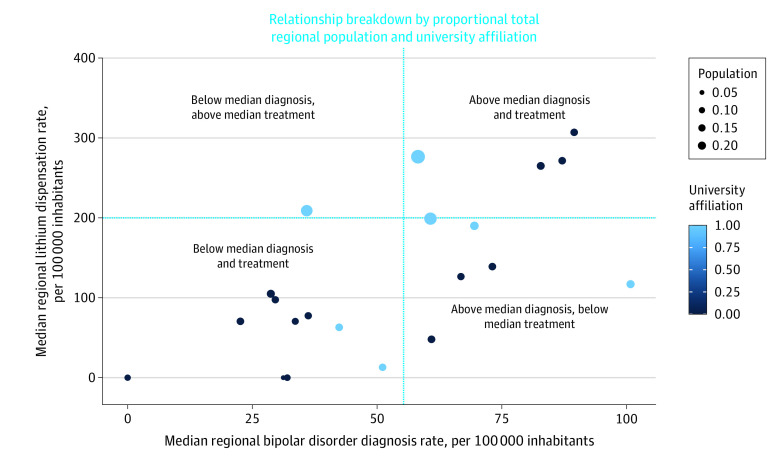
Median Regional Frequencies of Bipolar Disorder Diagnosis and Lithium Treatment in Male Adolescents Aged 15 to 19 Years, 2008-2021 Straight lines illustrate the national median for the same age group and period. University-affiliated counties are highlighted in blue, others in black. County population, retrieved from the Swedish Central Bureau of Statistics, is illustrated by circle diameter. There were large regional variations in bipolar disorder diagnosis frequencies and lithium dispensation rates in adolescent males. Median regional prevalence and prescription rates of bipolar disorder and lithium deviated over the national median by a factor of 0.00 to 1.82 for bipolar disorder and 0.00 to 1.53 for lithium treatment.

Across all 21 Swedish regions and both sexes, the mean (SD) prevalence rate of bipolar disorder diagnoses in adolescents aged 15 to 19 years was 100.4 (11.3) per 100 000 inhabitants. Female adolescents were almost 3 times more often diagnosed with bipolar disorder than male adolescents (mean [SD], 149.0 [19.6] vs 55.3 [6.1] per 100 000 inhabitants, respectively). Similarly, there were some national sex differences in the number of dispensations of lithium treatment among female and male adolescents (mean [SD], 326.9 [84.7] vs 199.6 [37.0] per 100 000 inhabitants, respectively). There were large regional variations in bipolar disorder diagnosis frequencies and lithium dispensation rates in adolescents across both sexes. Median regional prevalence and prescription rates of bipolar disorder and lithium deviated over the national median (measured across 2008-2021) by a factor of 0.46 to 2.61 and 0.00 to 1.82 for bipolar disorder diagnosis and 0.00 to 1.75 and 0.00 to 1.53 for lithium treatment in female and male adolescents, respectively (Figure 1 and Figure 2). There were substantial differences between the sexes regarding mean national suicide death rates across 2008 to 2021, with male adolescents overrepresented by a factor of approximately 2 (mean [SD], 5.3 [1.3] vs 9.1 [1.7] per 100 000 inhabitants for females and males, respectively). Similarly large deviations were observed regarding median regional ASM over the national median (factor of 0.00-2.16 for females and 0.00-2.91 for males), with some exceptions seemingly related to population size (ie, smaller-sized regions mainly exhibited median values of 0).

### Association of Bipolar Disorder Diagnosis Frequencies With Lithium Treatment Rates

The analysis between bipolar disorder diagnosis frequencies and lithium dispensation rates was performed separately in males and females. In this analysis, regional bipolar disorder diagnosis frequencies were negatively associated with lithium dispensation rates (β = −0.001; SE, 0.000; 95% CI, −0.001 to −0.000; *P* = .006) in males but not in females. In these analyses, bipolar disorder diagnosis frequencies were positively associated with PCAR in females (β = 0.120; SE, 0.049; 95% CI, 0.024-0.217; *P* = .02) but not in males (eTable 1 in Supplement 1). Diagnostic assessments of the final models did not show any significant problems (eFigures 3-5 in Supplement 1). Additional post hoc analyses were conducted using the same model as before but with the inclusion of a covariate measuring the regional annual number of participants who received at least 1 lithium prescription, which was included as an interaction term with Blom-normalized dispensation rates. The results of these analyses revealed an inverse association between regional bipolar disorder diagnosis frequencies and lithium dispensation rates (β = −0.162; 95% CI, −0.259 to −0.0640; *P* = .001). However, an independent and positive association was observed between bipolar diagnosis frequencies and lithium treatment rates (β = 0.008; SE, 0.001; 95% CI, 0.006-0.010; *P* < .001) (eTable 2 and eFigure 8 in Supplement 1).

### Association of Bipolar Disorder Diagnosis Frequencies With Suicide Death Rates 

Regional suicide death rates in adolescents were inversely associated with bipolar disorder diagnosis frequencies in males (β = −0.004; SE, 0.002; 95% CI, −0.0081 to −0.0004; *P* = .03), an association independent of lithium dispensation rates, an interaction term between diagnosis frequencies and dispensation rates, PCAR, OutInQuota, and an interaction term between PCAR and OutInQuota (Table 1). The associations between male bipolar disorder diagnosis frequencies and regional suicide death rates were found to be robust, even after adjusting for annual regional diagnosis rates of major depressive disorder and schizophrenia. Specifically, the results for the main variable (male bipolar disorder diagnosis frequencies) showed an inverse association with annual regional suicide mortality (β = −0.004; SE, 0.002; 95% CI, −0.008 to −0.000; *P* = .03), which was consistent across all analyses (eTable 3 in Supplement 1). Diagnostic assessments of the final models did not show significant problems (eFigures 1 and 6 in Supplement 1). In females, bipolar disorder diagnosis frequencies and lithium treatment utilization rates were not associated with suicide death rates (Table 1).

**Table 1.** Associations Between Adolescent Suicide Mortality, Bipolar Disorder Diagnosis Frequencies, and Lithium Dispensation Rates^a^

Variable	β	SE	*z* Value	*P* value
**Males**				
Intercept	2.545	0.146	17.481	<.001
Bipolar disorder diagnosis rate	−0.004	0.002	−2.182	.03
Lithium treatment rate^b^	−0.207	0.121	−1.717	.09
PCAR^b^	0.007	0.095	0.075	.94
OutInQuota^b^	−0.011	0.098	−0.114	.91
Bipolar disorder diagnosis rate:lithium treatment rate^b^	0.001	0.002	0.734	.46
PCAR^b^:OutInQuota^b^	−0.062	0.066	−0.948	.34
Random effects, conditional model^c^				
Year:region	Intercept	0.147	0.383	NA
Region	Intercept	1.58^E-08^	0	NA
**Females**				
Intercept	1.57	0.2	8.04	<.001
Bipolar disorder diagnosis rate^b^	0.17	0.11	1.55	.12
Lithium treatment rate^b^	0.20	0.11	1.76	.08
PCAR^b^	−0.07	0.15	−0.45	.65
OutInQuota^b^	−0.24	0.15	−1.62	.11
PCAR^b^:OutInQuota^b^	−0.07	0.09	−0.7	.48
Random effects, conditional model^c^				
Year:region	Intercept	0.081	0.284	NA
Region	Intercept	0.026	0.162	NA

Abbreviations: NA, not applicable; OutInQuota, reporting standard–adjusted proportion psychiatric visits to outpatient and inpatient facilities; PCAR, reporting standard–adjusted psychiatric care affiliation rate.

^a^ Number of observations, 294; year:region groups, 294; number of regions, 21. For males, dispersion parameter for Tweedie family, 8.66; Akaike information criterion, 1711.1; bayesian information criterion, 1751.6; log likelihood, −844.5; deviance, 1689.1, residual *df*, 283. For females, dispersion parameter for Tweedie family, 9.22; Akaike information criterion, 1168.1; bayesian information criterion, 1204; log likelihood, −574.0; deviance, 1148.1; residual *df*, 284.

^b^ Fixed-effects variables were subjected to transformation by the Blom method. The model did not evince any signs of bias from overdispersion or heteroscedasticity, and the 0 inflation assumption was confirmed as valid (eFigure 1 in Supplement 1).

^c^ Data presented are variance and SD.

In the validation analysis, quartile 4 ASM was inversely associated with bipolar disorder diagnosis frequencies (odds ratio [OR], 0.630; 95% CI, 0.457-0.869; *P* = .005) but unrelated to lithium dispensation rates, an interaction term between diagnosis frequencies and dispensation rates, PCAR, OutInQuota, and an interaction term between PCAR and OutInQuota (Table 2). This result is robust to the additional adjustment for annual regional diagnosis rates of major depressive disorder and schizophrenia, showing for the main variable an OR of 0.627 (95% CI, 0.458-0.869; *P* = .004), and consistently demonstrating a statistically significant inverse relationship between bipolar disorder diagnosis frequencies and quartile 4 ASM (eTable 4 in Supplement 1). Diagnostic assessments of the final models did not demonstrate any significant problems (eFigures 2 and 7 in Supplement 1).

**Table 2.** Associations in Males Between Top and IQR Observations of Adolescent Suicide Mortality (ASM) and Bipolar Disorder Diagnosis Frequencies and Lithium Dispensation Rates^a^

	β	SE	*z* Value	*P* value
Intercept	−0.953	0.173	−5.497	<.001
Bipolar disorder diagnosis rate^b^	−0.461	0.164	−2.816	.005
Lithium treatment rate^b^	−0.07	0.161	−0.434	.66
PCAR^b^	−0.057	0.203	−0.28	.78
OutInQuota^b^	0.052	0.204	0.252	.80
Bipolar disorder diagnosis rate^b^:lithium treatment rate^b^	0	0.171	0.819	.41
PCAR^b^:OutInQuota^b^	−0.286	0	−1.901	.06
Dispersion parameter for β-binomial (): 1	NA	NA	NA	NA
Random effects, conditional model^c^				
Year:region intercept^b^	5.51^E-08^	2.35^E-04^	NA	NA
Region intercept^b^	0.051	0.225	NA	NA

Abbreviations: NA, not applicable; OutInQuota, reporting standard–adjusted proportion of psychiatric visits to outpatient and inpatient facilities; PCAR, reporting standard–adjusted psychiatric care affiliation rates.

^a^ Number of observations, 294; year:region groups, 294; number of regions, 21.

^b^ Fixed-effects variables were subjected to transformation by Blom methods. The model did not evince any signs of bias from overdispersion or heteroscedasticity, and the 0 inflation assumption was confirmed as valid (eFigure 2 in Supplement 1).

^c^ Data presented are variance and SD.

## Discussion

The aim of this cross-sectional study was to investigate the possible presence of health inequities in regional diagnosis and lithium treatment rates of bipolar disorder in adolescents and to evaluate the association of early diagnosis of bipolar disorder with suicide prevention. The premise of the study is predicated on well-established concepts, such as a correlation of affective disorders with suicide, poor outcomes associated with early onset of bipolar disorder, significant delays in diagnosis of bipolar disorder (and subsequent treatment), and sex-based differences in suicide. In this retrospective, nationwide observational study during 2008-2021, substantial regional variations in bipolar disorder diagnosis frequencies among Swedish adolescents were found. Moreover, female adolescents were almost 3 times more often diagnosed with bipolar disorder. Regions with higher diagnosis rates of bipolar disorder in male adolescents also prescribed lithium to more patients but appear to be associated with lower dispensation rates. This could be interpreted to indicate that male adolescents may discontinue lithium treatment because of possible adverse effects or other issues with adherence to medication. Notably, bipolar disorder diagnosis frequencies were robustly associated with decreased suicide death rates in male adolescents unrelated to the regional annual number of lithium dispensations (exhibiting a nonsignificant trend in the same direction), depression and schizophrenia diagnosis rates, PCAR, and the proportion of psychiatry-related outpatient to inpatient visits. A diagnosis of bipolar disorder is estimated to be present in approximately 4.9% of unselected suicide cases in young adulthood.^15^ Our results were largely consistent with this estimate in that the main model findings estimated that an increase in male bipolar disorder diagnosis rates from 50 to 150 per 100 000 inhabitants would be associated with reductions in confirmed suicide death rates per 100 000 inhabitants by 0.43 (−0.00429 × 100), or approximately 4.7% of the mean national suicide death rate for male adolescents (0.43 / 9.1 × 100). This could be interpreted to indicate that factors associated with regional diagnosis rates of bipolar disorder may exert suicide-protective effects in male adolescents—ie, treatment efficacy, effects conferred from early diagnosis (and management), or other potential factors unaccounted for that may be associated with regional bipolar disorder diagnosis rates in youth.

### Strengths and Limitations

Strengths of this study include the use of fully replicable and openly available data^27,28,29,30^ from the Swedish National Board of Health and Welfare. This study encompassed all registered Swedish citizens aged 15 to 19 years between 2008 and 2021 and included 585 confirmed suicide deaths. Importantly, the sample size permitted the use of completed suicide as the variable of interest rather than less discriminatory secondary outcome variables such as attempted suicide,^17^ and substantial regional variations in bipolar disorder diagnosis rates made it possible to discern associations with suicide death rates at the regional level. Moreover, observed associations were independent of a plausible proxy variable for assessing regional annual PCAR, diagnosis rates of depression and schizophrenia, and the proportion of psychiatry-related outpatient to inpatient visits. This perceived strength of the study bears considerable relevance as regional variations in affiliation rates could be expected to positively influence local bipolar disorder diagnosis rates and simultaneously contribute to reduced suicide death rates.

The study also has several limitations. First, the observational data were aggregated, precluding a causal interpretation of the results. Second, the association between bipolar disorder diagnosis frequencies and adolescent suicide was significant only in males. Further studies are needed to elucidate whether these disparities are due to power issues, study design limitations, biological differences, or other factors. Confounding may also relate to potential overdiagnosis of bipolar disorder in female adolescents, as suggested by the observed discrepancies in female-to-male diagnosis ratios between this study (approximately 3:1) and reported findings from the US (approximately 1.27:1).^6^ Third, we could not ascertain received diagnoses or other clinical characteristics of adolescents who completed suicide. Validating the accuracy of bipolar disorder diagnoses in this population would have strengthened the study. However, the remarkably low prevalence of bipolar disorder in male adolescents in Sweden (national median over 2008-2021 of 0.06% vs an estimated 2.6% in the US^5,6^) suggests a cautious approach to bipolar disorder diagnosis in this population in Sweden, which may result in more accurate diagnoses in males. Nevertheless, external validation of our findings would be of value.

## Conclusions

In this cross-sectional study of a Swedish national registry covering the years 2008 to 2021 and encompassing 585 confirmed suicide deaths, regional diagnosis rates of bipolar disorder in adolescent males were associated with lower suicide death rates at an estimated magnitude of approximately 4.7% of the mean national suicide death rate. Results were independent of regional dispensation rates for lithium and PCAR and are consistent with reports estimating that a diagnosis of bipolar disorder is present in approximately 4.9% of suicide cases in young adulthood.^15^ Our findings could be associated with treatment efficacy, effects conferred from early diagnosis (and management), or other potential factors unaccounted for that may be associated with regional bipolar disorder diagnosis rates in youth and add to the literature indicating treatment gains from early diagnosis and management.

## Notes

Supplement 1.
eMethods.

eReferences.

eResults.
**eFigure 1.** Diagnostic Assessment of Main Model: DHARMa Test for Dispersion, QQ-Plots Residuals, and Residuals vs Predicted Lines (Males) **eFigure 2. **Diagnostic Assessment of Validation Model: DHARMa Test for Dispersion, QQ-Plots Residuals, and Residuals vs Predicted Lines (Males) **eFigure 3.** Diagnostic Assessment of Bipolar-Lithium Model: DHARMa Test for Dispersion, QQ-Plots Residuals and Residuals vs. Predicted Lines (Males) Before Correction **eFigure 4.** Diagnostic Assessment of Bipolar-Lithium Model: DHARMa Test for Dispersion, QQ-Plots Residuals, and Residuals vs Predicted Lines (Males) After Correction **eFigure 5.** Diagnostic Assessment of Validation Model: DHARMa Test for Dispersion, QQ-Plots Residuals, and Residuals vs Predicted Lines (Females) **eFigure 6.** Diagnostic Assessment of Post Hoc Analysis of Main Model (Including Depression and Schizophrenia Diagnosis Rates): DHARMa Test for Dispersion, QQ-Plots Residuals, and Residuals vs Predicted Lines (Males) **eFigure 7** Diagnostic Assessment of Post Hoc Analysis of Validation Model (Including Depression and Schizophrenia Diagnosis Rates): DHARMa Test for Dispersion, QQ-Plots Residuals, and Residuals vs Predicted Lines (Males) **eFigure 8.** Diagnostic Assessment of Post Hoc Bipolar-Lithium Model (Including the Regional Yearly Number of Unique Patients Receiving at Least 1 Lithium Prescription as a Covariate): DHARMa Test for Dispersion, QQ-Plots Residuals, and Residuals vs Predicted Lines (Males) **eTable 1.** Associations Between Lithium Utilization Rates and Bipolar Disorder Diagnosis Frequencies **eTable 2.** Post Hoc Analysis: Associations Between Lithium Utilization Rates, Number of Lithium-Treated Patients, and Bipolar Disorder Diagnosis Frequencies (Males) **eTable 3.** Post Hoc Analysis: Associations Between Adolescent Suicide Mortality, Bipolar Disorder and Major Depressive Disorder (MDD)/Schizophrenia Diagnosis Frequencies, and Lithium Utilization Rates (Males) **eTable 4.** Post Hoc Analysis: Associations in Males Between Adolescent Suicide Mortality, Bipolar Disorder and Major Depressive Disorder (MDD)/Schizophrenia Diagnosis Frequencies, and Lithium Utilization Rates, a Generalized Linear Mixed-Effects Model Modeled on the β-Binomial Distribution

Supplement 2.
Data Sharing Statement
