# Supplementary material for: Association of Bipolar Disorder Diagnosis With Suicide Mortality Rates in Adolescents in Sweden
Source: JAMA Psychiatry. 2023 May 24;80(8):796–802. doi: 10.1001/jamapsychiatry.2023.1390 (PMC10209824; doi:10.1001/jamapsychiatry.2023.1390)
Supplement: Supplement 1. — eMethods. eReferences. eResults. eFigure 1. Diagnostic Assessment of Main Model: DHARMa Test for Dispersion, QQ-Plots Residuals, and Residuals vs Predicted Lines (Males) eFigure 2. Diagnostic Assessment of Validation Model: DHARMa Test for Dispersion, QQ-Plots Residuals, and Residuals vs Predicted Lines (Males) eFigure 3. Diagnostic Assessment of Bipolar-Lithium Model: DHARMa Test for Dispersion, QQ-Plots Residuals and Residuals vs. Predicted Lines (Males) Before Correction eFigure 4. Diagnostic Assessment of Bipolar-Lithium Model: DHARMa Test for Dispersion, QQ-Plots Residuals, and Residuals vs Predicted Lines (Males) After Correction eFigure 5. Diagnostic Assessment of Validation Model: DHARMa Test for Dispersion, QQ-Plots Residuals, and Residuals vs Predicted Lines (Females) eFigure 6. Diagnostic Assessment of Post Hoc Analysis of Main Model (Including Depression and Schizophrenia Diagnosis Rates): DHARMa Test for Dispersion, QQ-Plots Residuals, and Residuals vs Predicted Lines (Males) eFigure 7 Diagnostic Assessment of Post Hoc Analysis of Validation Model (Including Depression and Schizophrenia Diagnosis Rates): DHARMa Test for Dispersion, QQ-Plots Residuals, and Residuals vs Predicted Lines (Males) eFigure 8. Diagnostic Assessment of Post Hoc Bipolar-Lithium Model (Including the Regional Yearly Number of Unique Patients Receiving at Least 1 Lithium Prescription as a Covariate): DHARMa Test for Dispersion, QQ-Plots Residuals, and Residuals vs Predicted Lines (Males) eTable 1. Associations Between Lithium Utilization Rates and Bipolar Disorder Diagnosis Frequencies eTable 2. Post Hoc Analysis: Associations Between Lithium Utilization Rates, Number of Lithium-Treated Patients, and Bipolar Disorder Diagnosis Frequencies (Males) eTable 3. Post Hoc Analysis: Associations Between Adolescent Suicide Mortality, Bipolar Disorder and Major Depressive Disorder (MDD)/Schizophrenia Diagnosis Frequencies, and Lithium Utilization Rates (Males) eTable 4. Post Hoc Analysis: Ass [file jamapsychiatry-e231390-s001.pdf]

Supplemental Online Content

Andersson P, Jokinen J, Jarbin H, Lundberg J, Desai Boström AE. Reduced regional suicide mortality association with bipolar disorder diagnosis rates in Swedish male adolescents. *JAMA Psychiatr*. Published online May 24, 2023. doi:10.1001/jamapsychiatry.2023.1390

eMethods.
eReferences.
eResults.
eFigure 1. Diagnostic Assessment of Main Model: DHARMa Test for Dispersion, QQ-Plots Residuals, and Residuals vs Predicted Lines (Males)
eFigure 2. Diagnostic Assessment of Validation Model: DHARMa Test for Dispersion, QQ-Plots Residuals, and Residuals vs Predicted Lines (Males)
eFigure 3. Diagnostic Assessment of Bipolar-Lithium Model: DHARMa Test for Dispersion, QQ-Plots Residuals and Residuals vs. Predicted Lines (Males) Before Correction
eFigure 4. Diagnostic Assessment of Bipolar-Lithium Model: DHARMa Test for Dispersion, QQ-Plots Residuals, and Residuals vs Predicted Lines (Males) After Correction
eFigure 5. Diagnostic Assessment of Validation Model: DHARMa Test for Dispersion, QQ-Plots Residuals, and Residuals vs Predicted Lines (Females)
eFigure 6. Diagnostic Assessment of Post Hoc Analysis of Main Model (Including Depression and Schizophrenia Diagnosis Rates): DHARMa Test for Dispersion, QQ-Plots Residuals, and Residuals vs Predicted Lines (Males)
eFigure 7 Diagnostic Assessment of Post Hoc Analysis of Validation Model (Including Depression and Schizophrenia Diagnosis Rates): DHARMa Test for Dispersion, QQ-Plots Residuals, and Residuals vs Predicted Lines (Males)
eFigure 8. Diagnostic Assessment of Post Hoc Bipolar-Lithium Model (Including the Regional Yearly Number of Unique Patients Receiving at Least 1 Lithium Prescription as a Covariate): DHARMa Test for Dispersion, QQ-Plots Residuals, and Residuals vs Predicted Lines (Males)
eTable 1. Associations Between Lithium Utilization Rates and Bipolar Disorder Diagnosis Frequencies
eTable 2. Post Hoc Analysis: Associations Between Lithium Utilization Rates, Number of Lithium-Treated Patients, and Bipolar Disorder Diagnosis Frequencies (Males)
eTable 3. Post Hoc Analysis: Associations Between Adolescent Suicide Mortality, Bipolar Disorder and Major Depressive Disorder (MDD)/Schizophrenia Diagnosis Frequencies, and Lithium Utilization Rates (Males)
eTable 4. Post Hoc Analysis: Associations in Males Between Adolescent Suicide Mortality, Bipolar Disorder and Major Depressive Disorder (MDD)/Schizophrenia Diagnosis Frequencies, and Lithium Utilization Rates, a Generalized Linear Mixed-Effects Model Modeled on the β-Binomial Distribution

This supplemental material has been provided by the authors to give readers additional information about their work.

© 2023 American Medical Association. All rights reserved.

eMethods.

2.1 Data sources, initial processing, and study design

This study was conducted according to STROBE (Strengthening The Reporting of Observational Studies in Epidemiology) guidelines[1], reporting on a nationwide sex-stratified observational study of suicide death rates, bipolar disorder diagnosis rates and lithium dispensation frequencies in 15-19-year-olds during 2008-2021. Data was retrieved for the 21 Swedish regions across 2008-2021 in the age-ranges 15-19 from the Swedish National Board of Health and Welfare[2] (freely available in Swedish [dataset][3–5]). Extracted data included registered bipolar disorder diagnosis frequencies (ICD-10: F31) in both specialized outpatient and inpatient care and confirmed suicide death rates (X60-X84) per 100,000 inhabitants, as well as the number of dispensations to adolescents recorded for lithium (ATC-code N05AN01) per 1,000 inhabitants in each region and age-group, respectively. In addition to the above, the regional yearly diagnosis frequencies of major depressive disorder (MDD; ICD-10: F32) and schizophrenia (ICD-10: F20) in 15-19-year-olds per 100,000 inhabitants were also included – values for these two variables were added for each region, year, and sex. Furthermore, the regional yearly number of 15-19-year-olds who received at least one prescription of lithium treatment for the year per 1,000 inhabitants was also obtained.

The following additional variables were retrieved to account for year-wise regional differences in reporting standards: the total number of registered visits and the total number of visits per 100,00 inhabitants without a registered diagnosis in both specialized outpatient care and inpatient care across all diagnoses (including somatic care), respectively; the unique number of patients with a registered psychiatric diagnosis (ICD-10: F00-F99) in both specialized outpatient care and inpatient care settings, respectively; and regional population size (subsequently calculated in percentage of the total national population). All data was extracted for males and females, separately. Values for lithium were multiplied by 100 to arrive at a representative estimate per 100,000 inhabitants. Data regarding diagnosis frequencies were available from 2008-2021, suicide death rate data from 1997 to 2021 and the number of patients receiving lithium treatment from 2006 to 2021. We excluded the years 1997-2007 from the analysis, as relevant information on diagnosis frequencies was not available for these years.

The sample thus encompassed aggregated data at the regional level representing all registered Swedish citizens that in the years 2008-2021 were aged 15-19-years-old who died by suicide or were diagnosed with BD (stratified by region and sex) – pertaining to 585 confirmed suicide deaths and 8,033 cases of BD. The aggregated data used for subsequent analyses entailed 588 unique observations, or 294 observations for each sex and variable (i.e., 21 regions, 14 years and two sexes). No data was excluded from downstream analyses.

2.2 Statistical considerations

2.2.1 Variable Distribution and Model Specifications

The distribution of the variables was investigated by Shapiro-wilks tests and visually inspected from histogram and Cullen and Frey plots[6,7]. No variable fully satisfied criteria for normal distribution. It was determined upon visual inspection of the semicontinuous nature of the response variable (many exact zeroes and continuous

© 2023 American Medical Association. All rights reserved.

positive outcomes) that this variable could be modeled on the Tweedie model of distributions – allowing for robust analysis of data with skewed distributions, and widely implemented in Generalized Linear Models[8]. For data exhibiting such characteristics, Favero et al. recently demonstrated the importance of considering random effects at the regional level[9]. Hence, it was determined that the analyses should be modelled on the tweedie distribution and that regional (and time) effects should be accounted for. We used glmmTMB models due to their increased flexibility in managing zero-inflated data and its higher speed when using multiple fixed effects as well as random effects[10].

2.2.2 Measures to reduce potential unmeasured confound

Confound could arise from region-specific effects regarding prevalence of bipolar disorder and suicide death rates in 15-19-year-olds. For example, regional variations in population size, socioeconomic status, substance abuse, or availability and quality of psychiatric care, could exert distorting effects on the extracted variables. Likewise, sex is a major confounder to both BD[11] and suicide death rates[12] and the model would not be sufficiently powered to adequately account for such complex interactions between BD, lithium and sex (as well as region and year). To reduce any distorting effects on our results from such potential sources of confound, generalized linear mixed effects models were implemented, designating BD and lithium as fixed effects, and region and year as random-intercept effects. Moreover, basing lithium values on the number of dispensations should reduce influence from observations of short-term treatment with lithium (compared to studying the number of individual patients receiving treatment), which was not considered conciliable with best-practice care. In summarizing, the data entailed sex-stratified regional rates (i.e., 21 regions, 14 years and 2 sexes = 588 total observations or 294 observations for each sex, respectively). Sex-stratified regional year-wise suicide death rates were defined as outcome variables, bipolar disorder diagnoses, lithium treatment and reporting-standard-adjusted psychiatric care affiliation rates (PCAR) and reporting-standard-adjusted proportions of psychiatric inpatient to outpatient visits (OutInQuota) constituted exposures, and year and region constituted random-intercept effect modifiers.

2.2.3 Analyses to reduce confound from regional year-wise variability in reporting standards and psychiatric care affiliation rates to inpatient and outpatient care settings

Reporting standards have improved over time – and may vary in outpatient and inpatient settings[2]. Moreover, regional year-wise variations in psychiatric care affiliation rates could exert endogenous confound – as high psychiatric care affiliation rates would be expected to be independently associated with both higher BD diagnosis rates and reduced suicide deaths. Moreover, it could be hypothesized that frequent psychiatry emergency department encounters in one area would not exert the same level of suicide-protection as regular outpatient psychiatric follow-up would in another. Therefore, the overall quality of psychiatric care could be driving the difference in suicide rates between these two areas, rather than frequency of diagnosis alone. Thus, it is important to account for putative confound from regional variations in reporting standards, psychiatric care affiliation rates and the relative proportion of inpatient to outpatient psychiatric visits over time[2]. Our method of adjusting for these factors is three-fold.

© 2023 American Medical Association. All rights reserved.

2.2.4 Reporting standards

To account for variations in reporting standards, the proportion of visits with a recorded diagnosis was calculated (i.e., 1 – (Number of visits without diagnosis code / Total number of visits)) across all diagnosis categories for each year and region. This was calculated separately for specialized outpatient care and inpatient care. Therefore, this value constituted the best-available estimate of the overall number of registered visits with a recorded diagnosis for each region and year. These data were not available specifically for psychiatric care. Nevertheless, given substantial regional and yearly variations, it was considered reasonable to assume that region and year-specific variations in reporting standards of psychiatric diagnoses developed similarly to that which was observed across all diagnosis categories. Moreover, as youth BD necessitating inpatient treatment upon discharge require frequent follow-ups in outpatient care, it could be hypothesized that patients receiving BD diagnoses in inpatient care would also contribute the identical diagnosis in outpatient care settings. Therefore, adjusting BD diagnosis rates for reporting standards in outpatient care settings was considered more appropriate for estimating the total frequency of yearly regional BD diagnosis frequencies (compared to reporting standards for inpatient care). Thus, recorded BD diagnosis rates were adjusted to this variable to account for lapses of potentially unreported diagnoses (i.e., (recorded BD diagnosis rate / proportion of visits to outpatient care with registered diagnosis across all diagnosis categories).

2.2.5 Psychiatric care affiliation rates

Mental health shortage areas have been previously associated with increases in suicide rates[13]. To account for regional year-wise variations in psychiatric care affiliation rates, the unique number of patients with a registered psychiatric diagnosis (ICD-10: F00-F99) in specialized inpatient and outpatient care settings were retrieved. The number of patients receiving outpatient and inpatient care across all psychiatric diagnosis categories, could also be biased from variations in reporting standards. Moreover, reporting standards may be different between inpatient and outpatient care for the same region and year. Thus, these variables were adjusted by the same estimate as derived in Section 2.2.1.1 for each region and year separately (i.e., (recorded psychiatric diagnoses in outpatient care settings / proportion of visits with registered diagnoses in outpatient settings across all diagnosis categories) and (recorded psychiatric diagnoses in inpatient care settings / proportion of visits with registered diagnoses in inpatient settings across all diagnosis categories).

2.2.6 Relative proportion of psychiatric outpatient to inpatient visits

The relative proportion of psychiatric outpatient to inpatient visits could influence confirmed suicide death rates[14]. To reduce this potential source of bias, the proportion of reporting-standard-adjusted psychiatric care affiliation rate variables in outpatient and inpatient care were calculated by division of the latter to the former – representing the proportion of reporting-standard adjusted patients receiving outpatient to inpatient care.

2.2.6 Examination of the potential impact of MDD and schizophrenia diagnosis rates on regional suicide death rates in adolescents

© 2023 American Medical Association. All rights reserved.

To examine the potential impact of other psychiatric diagnoses on suicide death rates in adolescents, we included the combined regional yearly diagnosis rates of major depressive disorder (MDD; ICD-10 code: F32) and schizophrenia (ICD-10 code: F20) for the same age group as a fixed-effects variable in significant models. We adjusted the MDD/schizophrenia diagnosis rate variable for yearly regional variations in reporting standards using the method described in section 2.2.4, which is consistent with the methods used to adjust BD diagnosis rates. These post-hoc analyses were designed to assess the assumption that alterations in suicide death rates are specifically associated with changes in yearly regional prevalence rates of BD and not due to changes in MDD or schizophrenia diagnosis rates, which could introduce endogenous confounding.

2.3 Statistical Analysis

2.3.1 Descriptive figures

To illustrate regional variations, regional median values for 2008-2021 – pertaining to BD diagnosis and lithium treatment rates - were illustrated in x-y-scatterplots. As a second step, treatment and diagnosis variables were investigated for collinearity in each sex-group, separately. The ‘glmmTMB’ package for R-statistics was implemented for these analyses[15], modeled on the Tweedie-distribution[8].

2.3.2 Model diagnostics

Significant models were tested for dispersion and heteroscedasticity. First, we used the DHARMa test for dispersion (‘testDispersion’-function of the DHARMa package for R Statistics[16]) to test if main models were under- or overdispersed. Residuals were illustrated in ‘QQ-plots’ and ‘Residuals vs. predicted values’ plots using the ‘simulateResiduals’-function of the DHARMa[16]. The exact p-values for the quantile lines in the plot were calculated by the ‘testQuantiles’-function of the same R package. Diagnostic assessment of the main model did not demonstrate any significant problems (**Supplemental Figure 1.**). The validation model was not demonstrated as over- or underdispersed and did not show any signs of significant heteroscedasticity (**Supplemental Figure 2.**). The association analysis between bipolar diagnosis rates and lithium dispensation frequencies exhibited signs of heteroscedasticity in the case of males (**Supplemental Figure 3.**) but not females (**Supplemental Figure 5.**). A simple overdispersion correction was added by adding an interaction term between region and regional population weights, after which the model exhibited no signs of over- or underdispersion, or heteroscedasticity (**Supplemental Figure 4.**).

2.3.3 Additional Post-hoc analyses

To provide further insight into the association between bipolar diagnosis rates and lithium dispensation rates, additional post-hoc analyses were conducted. These analyses involved adjusting for the number of patients who received at least one lithium prescription per region and year, which was included as an interaction term with dispensation rates. The analyses were performed to account for any potential confounding effects that may have impacted the observed relationship between bipolar diagnosis and lithium dispensation rates. To avoid violating model assumptions, the model was specified as zero-inflated on the added covariate representing the participants who received at least one lithium prescription per region and year.

© 2023 American Medical Association. All rights reserved.

Furthermore, both the main and validation analyses were re-performed post-hoc with the inclusion of a variable measuring the combined reporting-standard adjusted regional diagnosis rates of major depressive disorder (ICD-10: F32) and schizophrenia (ICD-10: F20). The addition of this variable was aimed at providing a more comprehensive understanding of the relationship between bipolar disorder and suicide risk reduction, by accounting for potential confounding factors that may have influenced the observed relationship.

All post-hoc analyses were tested for dispersion and heteroscedasticity (**Supplemental Figure 6**., **Supplemental Figure 7., Supplemental Figure 8**). These statistical checks were performed to ensure the robustness and validity of the results obtained from the analyses.

eReferences.

1. Elm E von, Altman DG, Egger M, Pocock SJ, Gøtzsche PC, Vandenbroucke JP. Strengthening the Reporting of Observational Studies in Epidemiology (STROBE) statement: guidelines for reporting observational studies. *BMJ*. 2007;335(7624):806-808. doi:10.1136/BMJ.39335.541782.AD
2. Ludvigsson JF, Andersson E, Ekbom A, et al. External review and validation of the Swedish national inpatient register. *BMC Public Health*. 2011;11(1):1-16. doi:10.1186/1471-2458-11-450/COMMENTS
3. Stockholm. Statistikområden, Dödsorsaker [internet]. Socialstyrelsen. Accessed September 24, 2022. https://www.socialstyrelsen.se/statistik-och-data/statistik/statistikdatabasen
4. Stockholm. Socialstyrelsen, Läkemedel [internet]. Socialstyrelsen. Accessed September 24, 2022. https://www.socialstyrelsen.se/statistik-och-data/statistik/statistikdatabasen/
5. Stockholm. Socialstyrelsen, Diagnoser i sluten vård och specialiserad öppenvård [internet]. Socialstyrelsen. Accessed September 24, 2022. https://www.socialstyrelsen.se/statistik-och-data/statistik/statistikdatabasen/
6. Delignette-Muller ML, Dutang C. fitdistrplus: An R Package for Fitting Distributions. *J Stat Softw*. 2015;64(4):1-34. doi:10.18637/JSS.V064.I04
7. Cullen AC, Frey HC. *Probabilistic Techniques in Exposure Assessment: A Handbook for Dealing with ... - Alison C. Cullen, H. Christopher Frey - Google Књиге*. Plenum Press; 1999. Accessed September 4, 2022. https://link.springer.com/book/9780306459566
8. Ctr S, Glickman ME, Ctr S, Hours O, Branson Z. *Statistics 244 Linear and Generalized Linear Models*.; 2011. Accessed September 4, 2022. https://www.routledge.com/Generalized-Linear-Models/McCullagh-Nelder/p/book/9780412317606
9. Fávero LP, Hair JF, Souza R de F, Albergaria M, Brugni T V. Zero-inflated generalized linear mixed models: A better way to understand data relationships. *Mathematics*. 2021;9(10):1100. doi:10.3390/MATH9101100/S1

© 2023 American Medical Association. All rights reserved.

10. Brooks ME, Kristensen K, van Benthem KJ, et al. glmmTMB balances speed and flexibility among packages for zero-inflated generalized linear mixed modeling. *R J*. 2017;9(2):378-400. doi:10.32614/RJ-2017-066

11. Duax JM, Youngstrom EA, Calabrese JR, Findling RL. Sex differences in pediatric bipolar disorder. *J Clin Psychiatry*. 2007;68(10):1565-1573. doi:10.4088/JCP.V68N1016

12. Bridge JA, Goldstein TR, Brent DA. Adolescent suicide and suicidal behavior. *J Child Psychol Psychiatry*. 2006;47(3-4):372-394. doi:10.1111/J.1469-7610.2006.01615.X

13. Ku BS, Li J, Cathy Lally, Compton MT, Druss BG. Associations between mental health shortage areas and county-level suicide rates among adults aged 25 and older in the USA, 2010 to 2018. *Gen Hosp Psychiatry*. 2021;70:44-50. doi:10.1016/J.GENHOSPPSYCH.2021.02.001

14. Chung DT, Ryan CJ, Hadzi-Pavlovic D, Singh SP, Stanton C, Large MM. Suicide Rates After Discharge From Psychiatric Facilities: A Systematic Review and Meta-analysis. *JAMA psychiatry*. 2017;74(7):694-702. doi:10.1001/JAMAPSYCHIATRY.2017.1044

15. Brooks ME, Kristensen K, Benthem KJ van, et al. Modeling zero-inflated count data with glmmTMB. *bioRxiv*. Published online May 1, 2017:132753. doi:10.1101/132753

16. DHARMa: residual diagnostics for hierarchical (multi-level/mixed) regression models. Accessed December 18, 2022. https://cran.r-project.org/web/packages/DHARMa/vignettes/DHARMa.html

17. Ludwig O. Blom, Gunnar: Statistical estimates and transformed beta-variables. Wiley/New York, Almquist und Wiksell/Stockholm 1958; 176 S., Kr. 20,—. *Biom Z*. 1961;3(4):285-285. doi:10.1002/BIMJ.19610030410

© 2023 American Medical Association. All rights reserved.

eResults.

2.1 Baseline Characteristics of Data

Across the 21 Swedish regions, the median prevalence rate of reporting-standard adjusted diagnoses of major depressive disorder and schizophrenia per 100,000 inhabitants in 15-19-year-olds from 2008 to 2021 was 1990.7 (SD: 415.5) in females and 730.4 (SD: 133.0) in males.

© 2023 American Medical Association. All rights reserved.

eFigure 1. Diagnostic Assessment of Main Model: DHARMa Test for Dispersion, QQ-Plots Residuals, and Residuals vs Predicted Lines (Males)

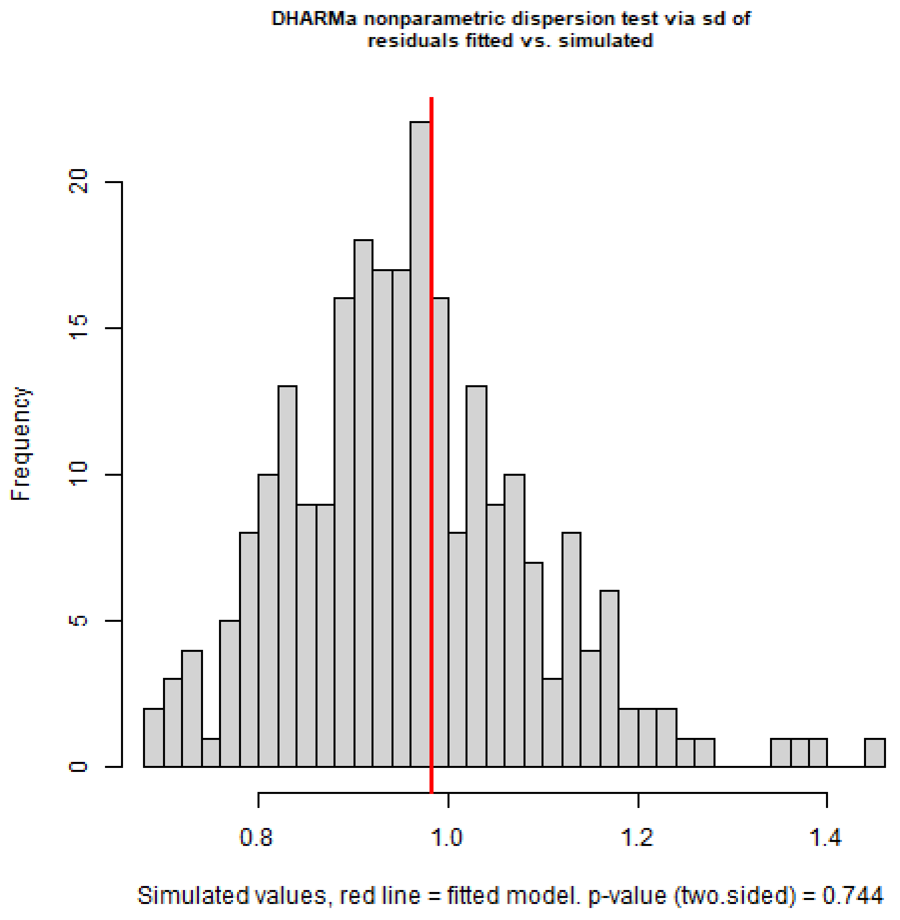

© 2023 American Medical Association. All rights reserved.

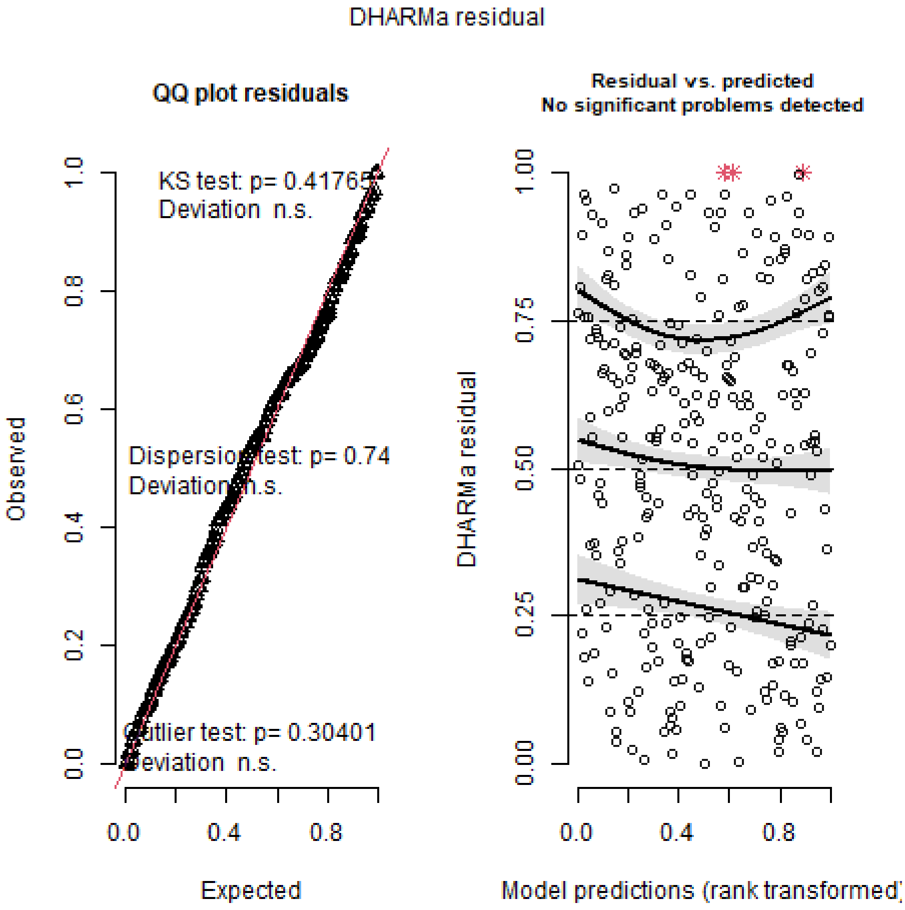

Legend: The association analyses between adolescent suicide mortality rates and BD diagnosis frequencies and suicide death rates were investigated in males, applying generalized linear mixed effects models modeled on the Tweedie-distribution whereby an interaction term between the yearly regional BD diagnosis frequencies and the yearly regional number of lithium dispensations was designated as a fixed-effects variable. An interaction term between PCAR and OutInQuota were further included as independent fixed-effects variables. Region and year were included as random-intercept effects. Fixed effects variables (excluding BD diagnosis frequencies) were subjected to transformation by Blom's methods. This model was tested post-hoc for dispersion and heteroscedasticity – demonstrating no such signs [illustrated in this figure].
Abbreviations: PCAR, reporting-standard-adjusted psychiatric care affiliation rates; OutInQuota, the proportion of reporting-standard-adjusted psychiatric visits to outpatient and inpatient facilities.

© 2023 American Medical Association. All rights reserved.

eFigure 2. Diagnostic Assessment of Validation Model: DHARMa Test for Dispersion, QQ-Plots Residuals, and Residuals vs Predicted Lines (Males)

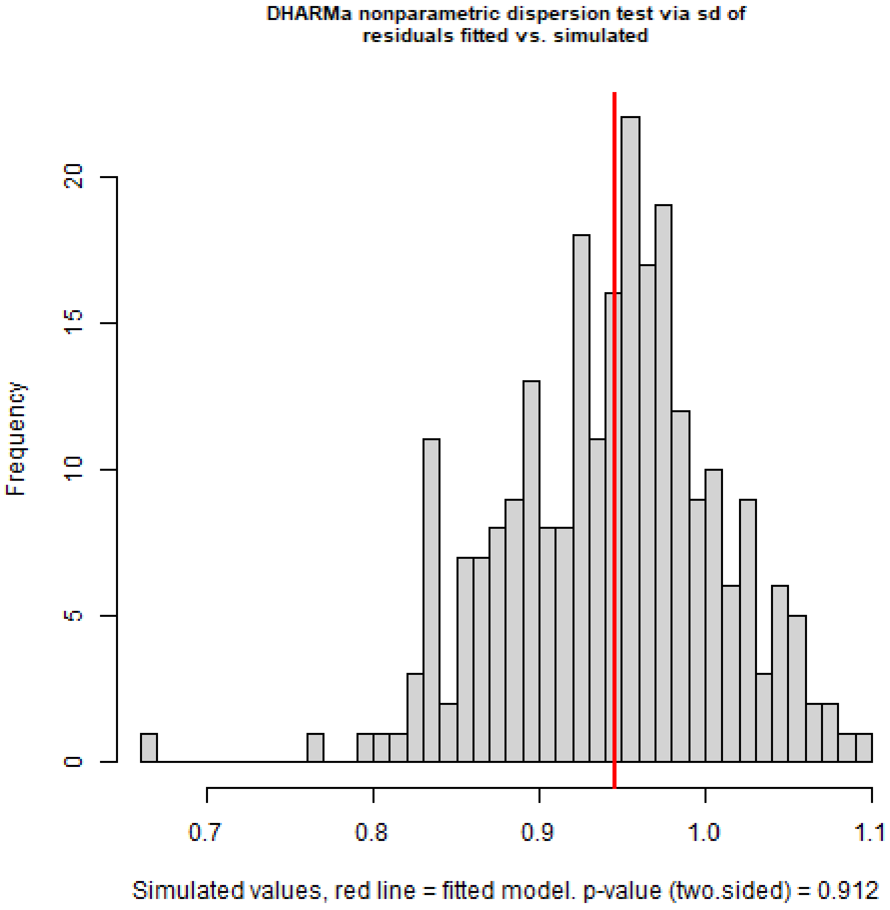

© 2023 American Medical Association. All rights reserved.

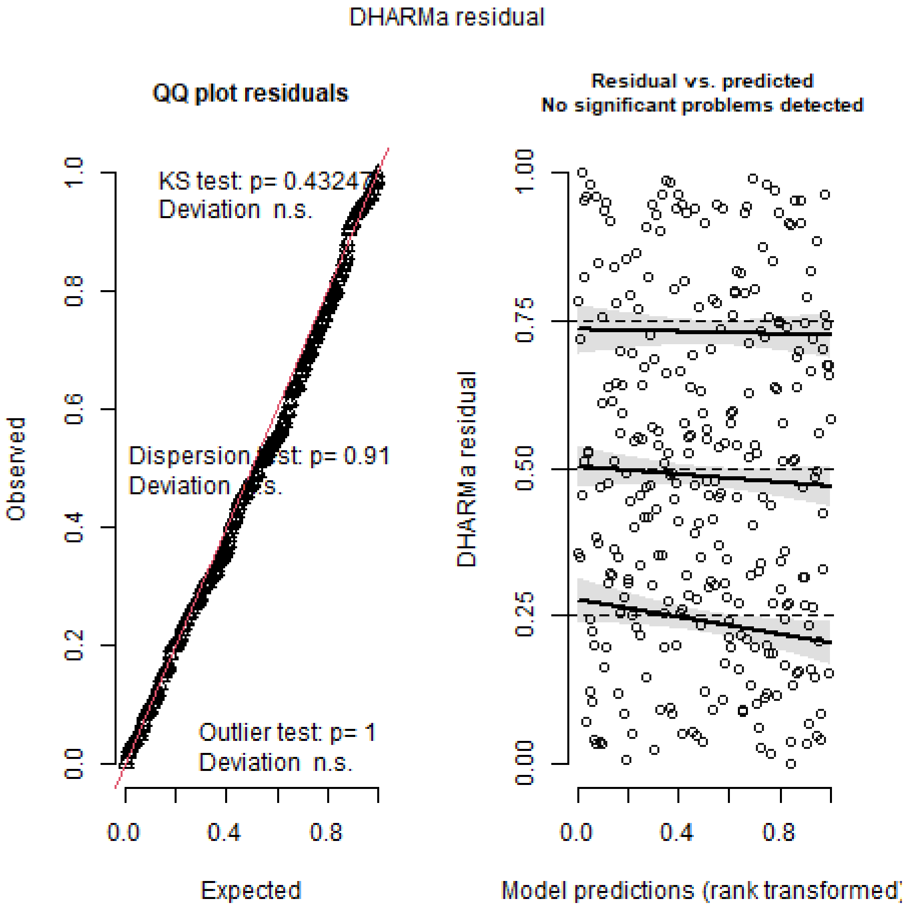

Legend: ASM was dichotomized based on the 75th percentile – whereby top-quartile (Q4) observations regarding ASM were compared to lower-quartile (Q1-Q3) observations. The association analyses between dichotomized adolescent suicide mortality rates and BD diagnosis frequencies were investigated in males, applying generalized linear mixed effects models modeled on the beta-binomial distribution whereby an interaction term between the yearly regional BD diagnosis frequencies and the yearly regional number of lithium dispensations was designated as an independent fixed-effects variable. An interaction term between PCAR and OutInQuota were further included as an independent fixed-effects variable. Region and year were included as random-intercept effects. All fixed effects variables were subjected to transformation by Blom's methods. This model was tested post-hoc for dispersion and heteroscedasticity – demonstrating no such signs [illustrated in this figure].
Abbreviations: PCAR, reporting-standard-adjusted psychiatric care affiliation rates; OutInQuota, the proportion of reporting-standard-adjusted psychiatric visits to outpatient and inpatient facilities.

© 2023 American Medical Association. All rights reserved.

eFigure 3. Diagnostic Assessment of Bipolar-Lithium Model: DHARMa Test for Dispersion, QQ-Plots Residuals and Residuals vs. Predicted Lines (Males) Before Correction

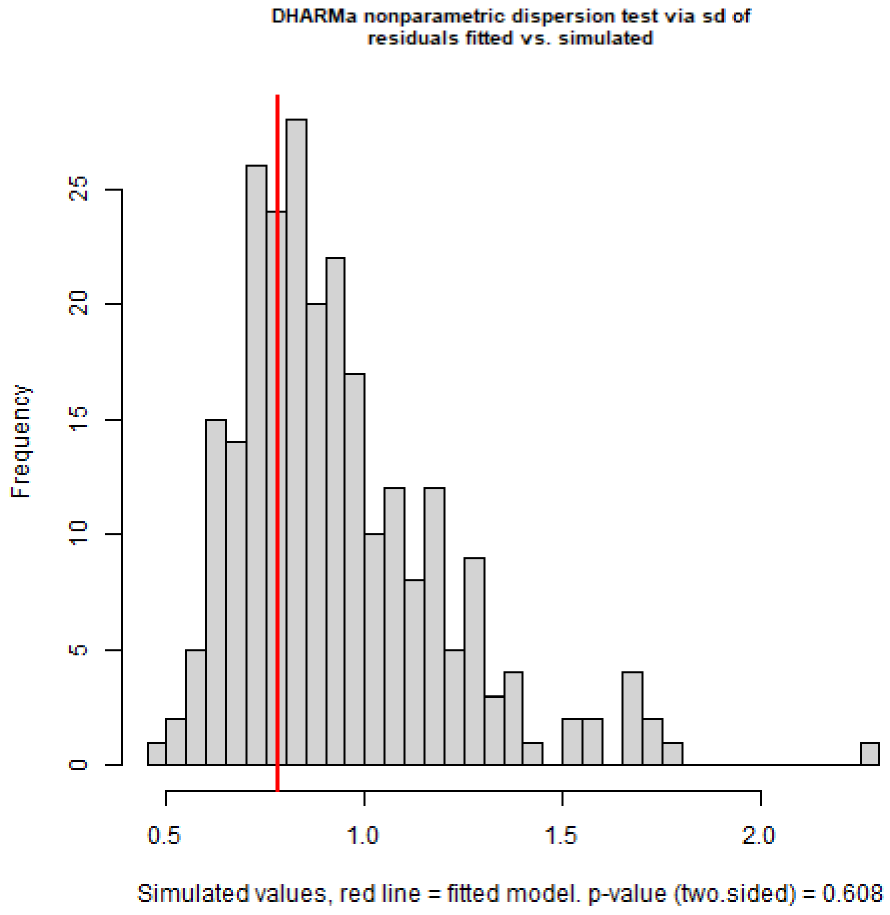

© 2023 American Medical Association. All rights reserved.

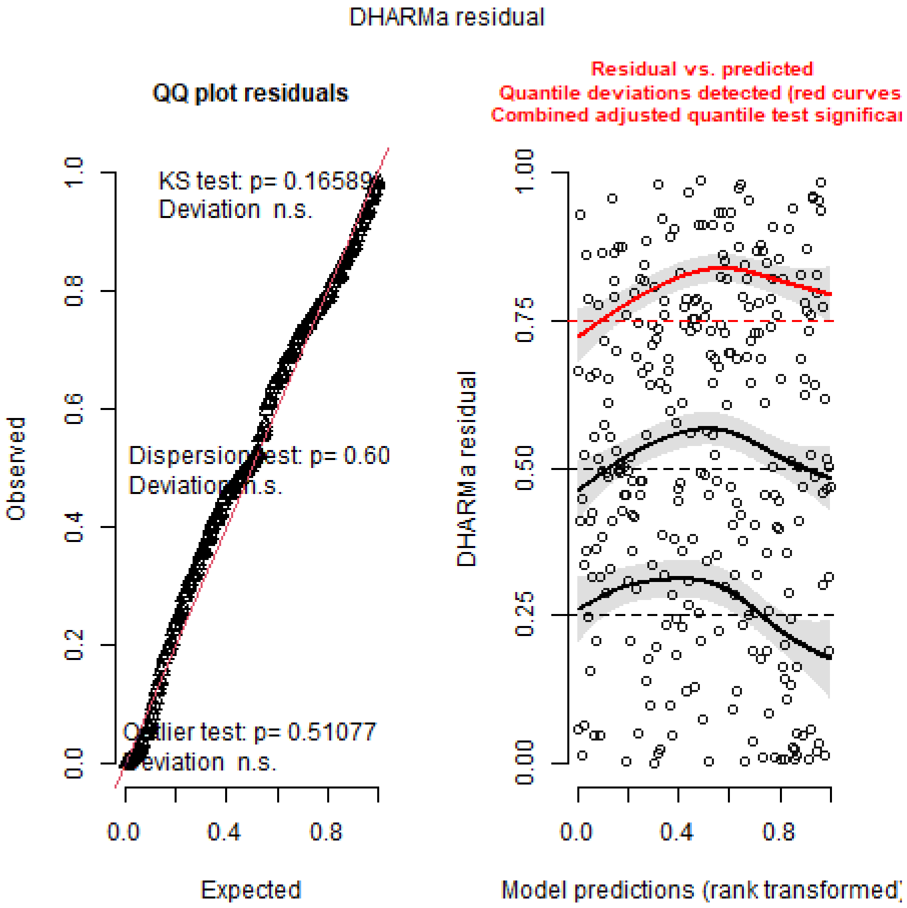

Legend: Associations between the outcome variable (bipolar diagnosis rates), exposures (lithium dispensation frequencies and an interaction term between PCAR and OutinQuota) were investigated in males by generalized linear mixed effects models, whereby exposure variable were designated as independent fixed-effects variables. Random-intercept effect modifiers pertained to region and year. In this analysis, the following variables were subjected to normalization by Blom's method[17] to avoid violating model assumptions: PCAR and OutInQuota. This model was tested post-hoc for dispersion and heteroscedasticity – demonstrating significant signs of heteroskedasticity [illustrated in this figure].

Abbreviations: PCAR, reporting-standard-adjusted psychiatric care affiliation rates; OutInQuota, the proportion of reporting-standard-adjusted psychiatric visits to outpatient and inpatient facilities.

© 2023 American Medical Association. All rights reserved.

eFigure 4. Diagnostic Assessment of Bipolar-Lithium Model: DHARMa Test for Dispersion, QQ-Plots Residuals, and Residuals vs Predicted Lines (Males) After Correction

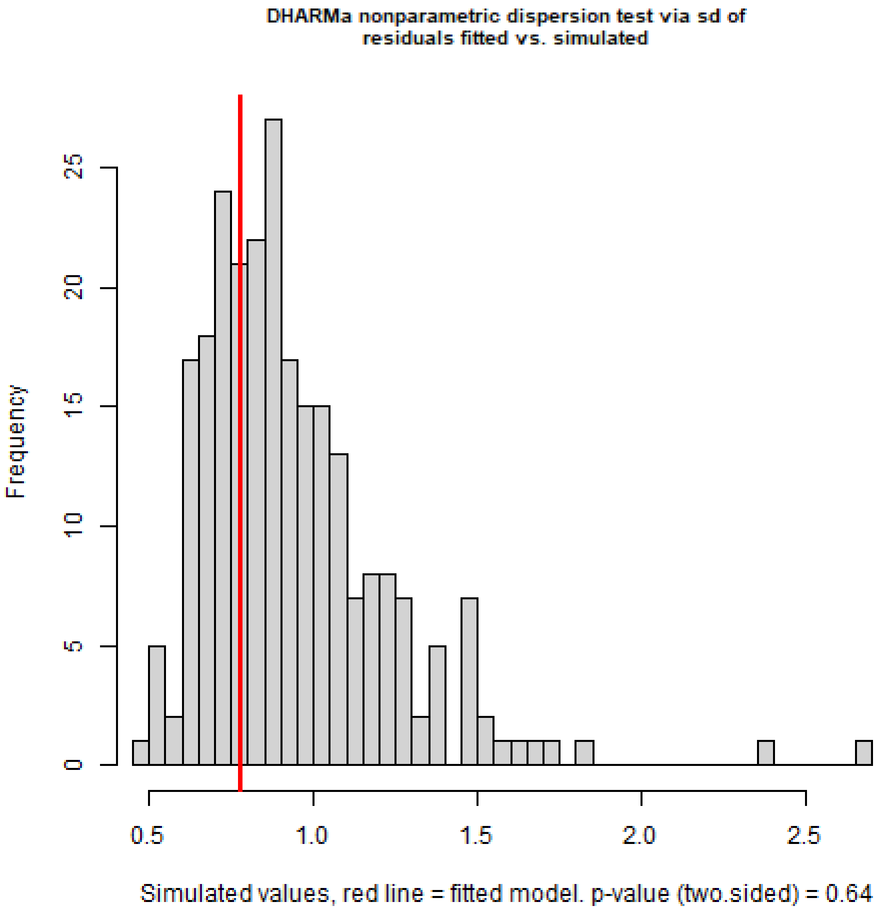

© 2023 American Medical Association. All rights reserved.

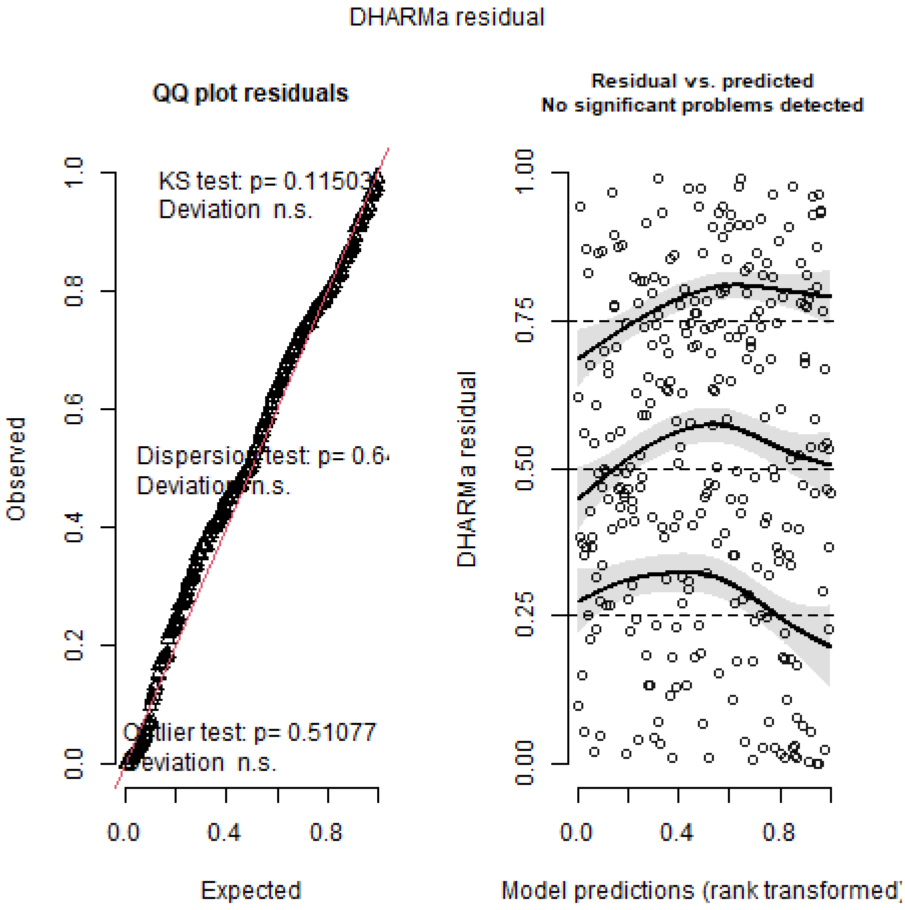

Legend: Associations between the outcome variable (bipolar diagnosis rates), exposures (lithium dispensation frequencies and an interaction term between PCAR and OutinQuota) were investigated in males by generalized linear mixed effects models, whereby exposure variable were designated as independent fixed-effects variables. Random-intercept effect modifiers pertained to year and an interaction term between region and proportional regional population size [the latter interaction term was added as a simple overdispersion correction]. In this analysis, the following variables were subjected to normalization by Blom's method[17] to avoid violating model assumptions: PCAR and OutInQuota. This model was tested post-hoc for dispersion and heteroscedasticity – demonstrating no such signs [illustrated in this figure].

Abbreviations: PCAR, reporting-standard-adjusted psychiatric care affiliation rates; OutInQuota, the proportion of reporting-standard-adjusted psychiatric visits to outpatient and inpatient facilities.

© 2023 American Medical Association. All rights reserved.

eFigure 5. Diagnostic Assessment of Validation Model: DHARMa Test for Dispersion, QQ-Plots Residuals, and Residuals vs Predicted Lines (Females)

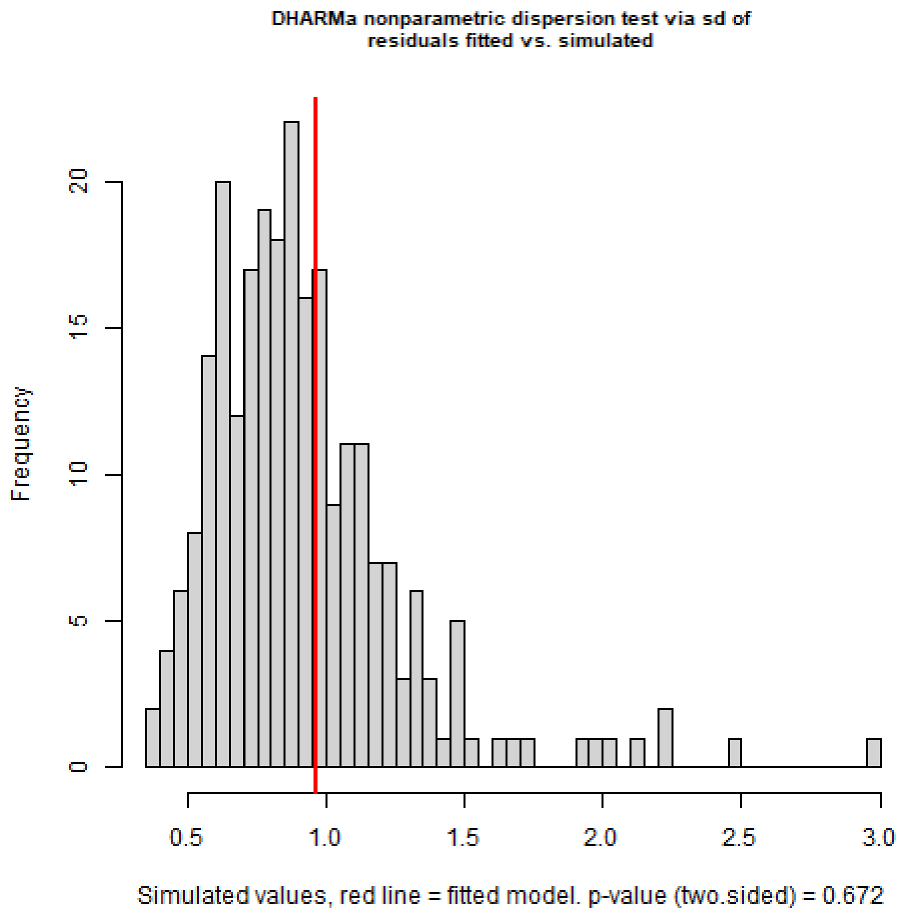

© 2023 American Medical Association. All rights reserved.

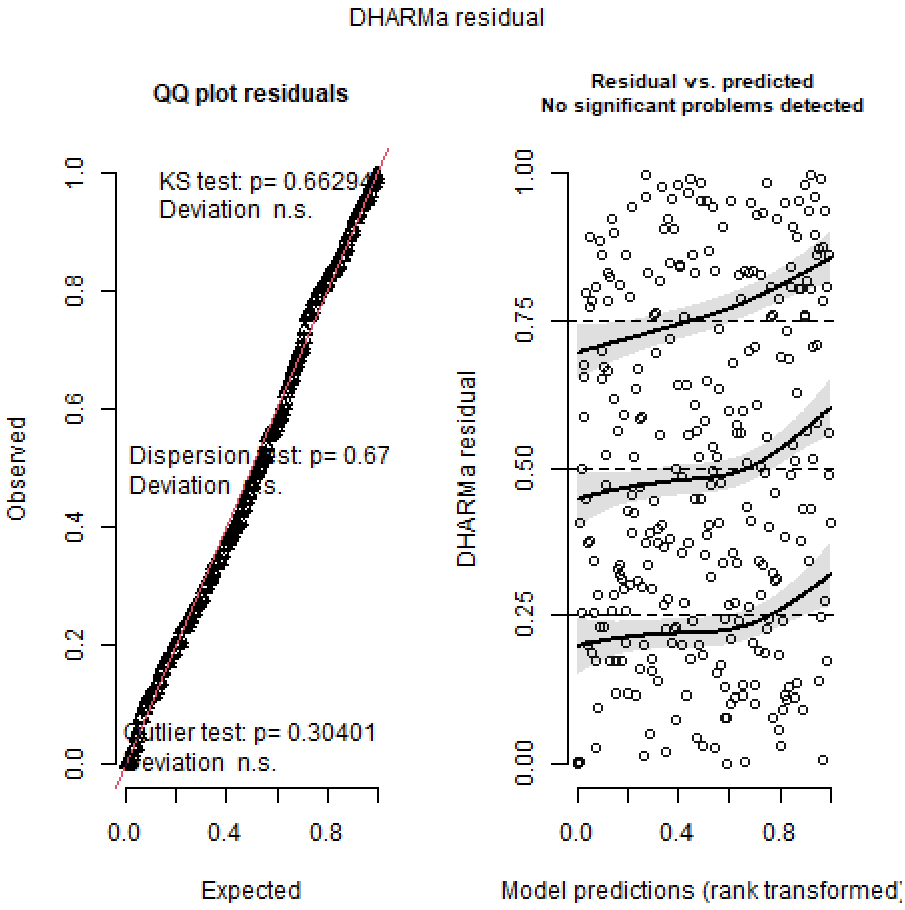

Legend: Associations between the outcome variable (bipolar diagnosis rates), exposures (lithium dispensation frequencies and an interaction term between PCAR and OutInQuota) were investigated in females by generalized linear mixed effects models, whereby exposure variable were designated as independent fixed-effects variables. Random-intercept effect modifiers pertained to region and year. In this analysis, the following variables were subjected to normalization by Blom's method[17] to avoid violating model assumptions: Lithium dispensation rates, PCAR and OutInQuota. This model was tested post-hoc for dispersion and heteroscedasticity - evincing no such signs [illustrated in this figure].
Abbreviations: PCAR, reporting-standard-adjusted psychiatric care affiliation rates; OutInQuota, the proportion of reporting-standard-adjusted psychiatric visits to outpatient and inpatient facilities.

© 2023 American Medical Association. All rights reserved.

eFigure 6. Diagnostic Assessment of Post Hoc Analysis of main Model (Including Depression and Schizophrenia Diagnosis Rates): DHARMa Test for Dispersion, QQ-Plots Residuals, and Residuals vs Predicted Lines (Males)

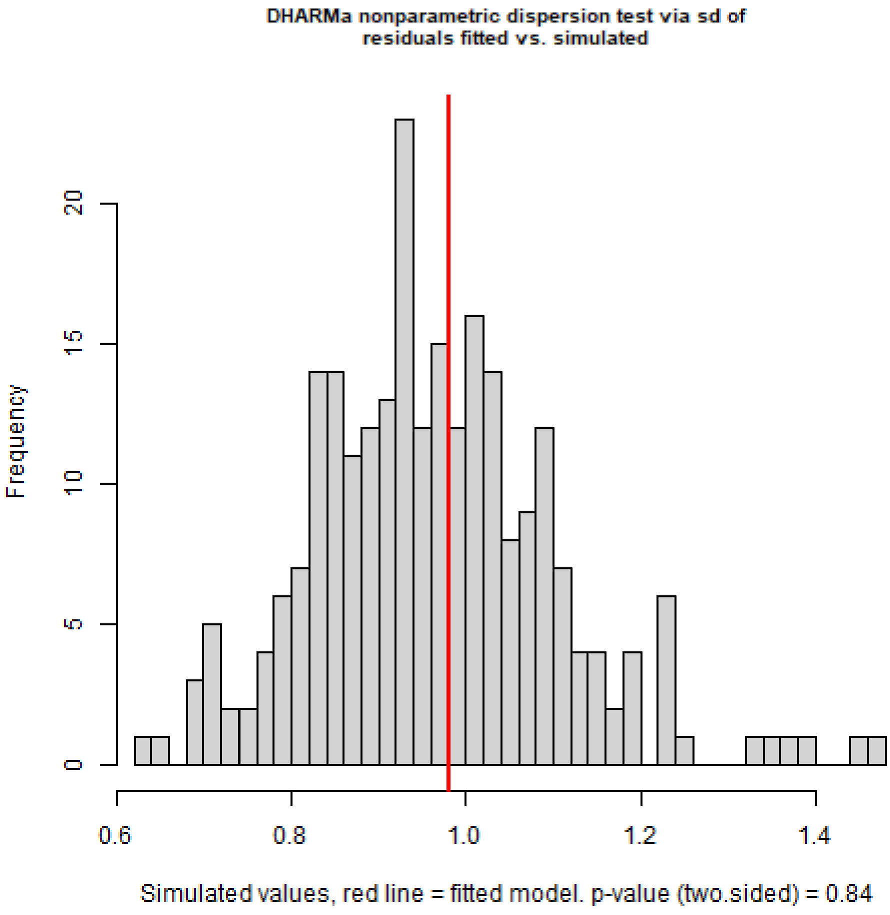

© 2023 American Medical Association. All rights reserved.

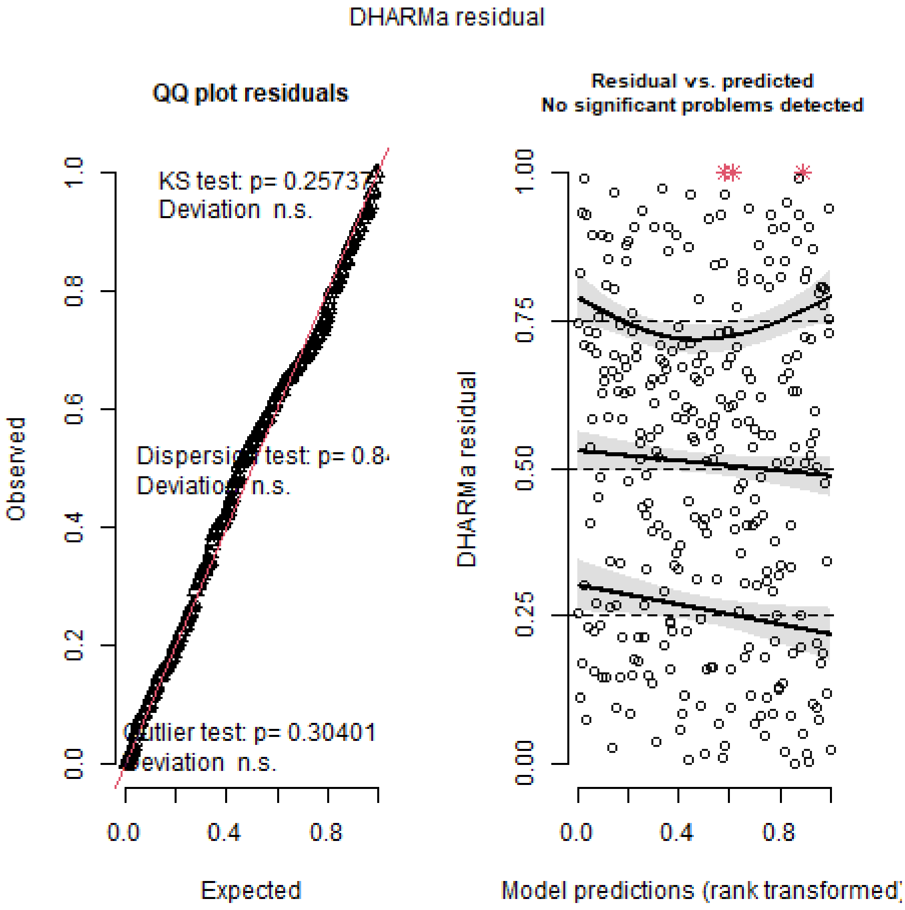

Legend: The association analyses between adolescent suicide mortality rates and BD diagnosis frequencies were investigated in males, applying generalized linear mixed effects models modeled on the Tweedie-distribution whereby an interaction term between the yearly regional BD diagnosis frequencies and the yearly regional number of lithium dispensations was designated as a fixed-effects variable. An interaction term between PCAR and OutInQuota were further included as independent fixed-effects variables. An additional fixed-effects variable was the regional yearly combined major depressive disorder (MDD; ICD-10: F32) and schizophrenia (ICD-10: F20) diagnosis rates. Region and year were included as random-intercept effects. Fixed effects variables (excluding BD diagnosis frequencies) were subjected to transformation by Blom's methods. This model was tested post-hoc for dispersion and heteroscedasticity – demonstrating no such signs [illustrated in this figure].
Abbreviations: PCAR, reporting-standard-adjusted psychiatric care affiliation rates; OutInQuota, the proportion of reporting-standard-adjusted psychiatric visits to outpatient and inpatient facilities.

© 2023 American Medical Association. All rights reserved.

eFigure 7. Diagnostic Assessment of Post Hoc Analysis of Validation Model (Including Depression and Schizophrenia Diagnosis Rates): DHARMa Test for Dispersion, QQ-Plots Residuals, and Residuals vs Predicted Lines (Males)

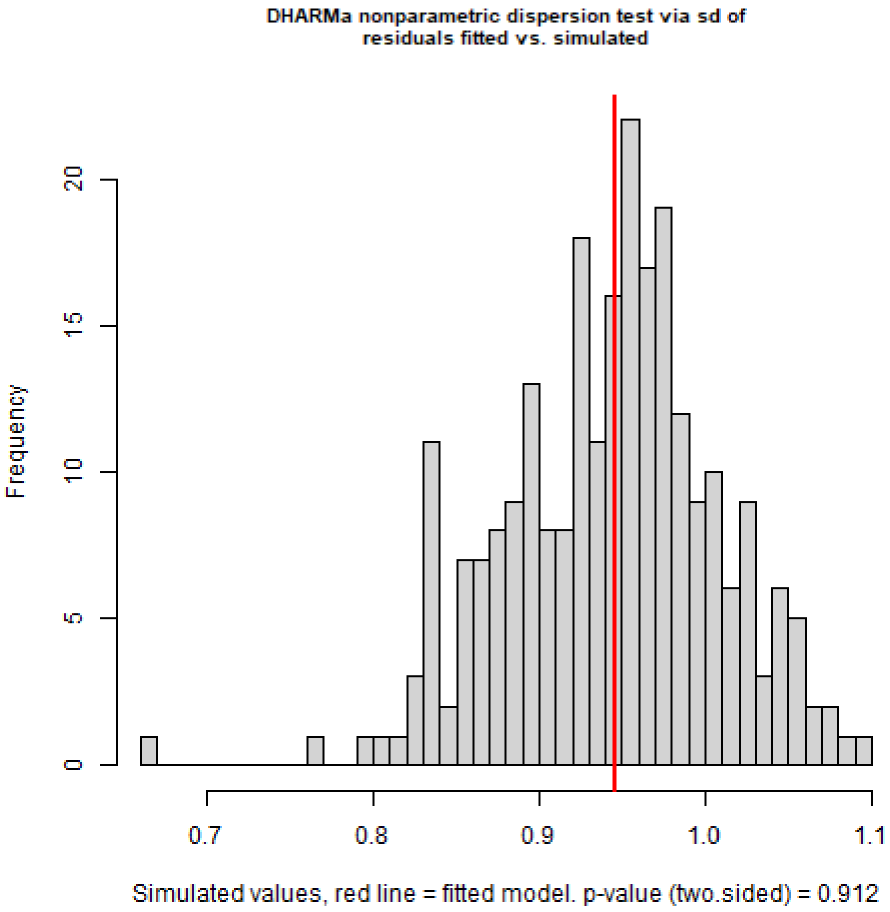

© 2023 American Medical Association. All rights reserved.

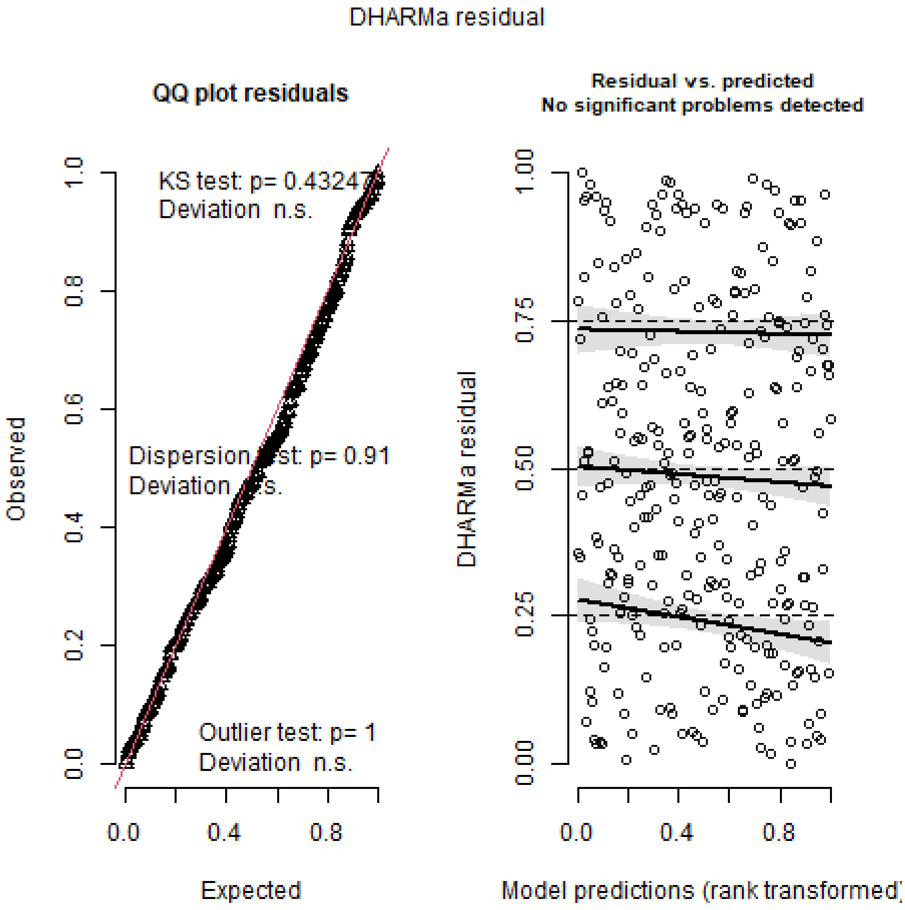

Legend: ASM was dichotomized based on the 75th percentile – whereby top-quartile (Q4) observations regarding ASM were compared to lower-quartile (Q1-Q3) observations. The association analyses between dichotomized adolescent suicide mortality rates and BD diagnosis frequencies were investigated in males, applying generalized linear mixed effects models modeled on the beta-binomial distribution whereby an interaction term between the yearly regional BD diagnosis frequencies and the yearly regional number of lithium dispensations was designated as an independent fixed-effects variable. An interaction term between PCAR and OutInQuota were further included as an independent fixed-effects variable. An additional fixed-effects variable was the regional yearly combined major depressive disorder (MDD; ICD-10: F32) and schizophrenia (ICD-10: F20) diagnosis rates. Region and year were included as random-intercept effects. All fixed effects variables were subjected to transformation by Blom's methods. This model was tested post-hoc for dispersion and heteroscedasticity – demonstrating no such signs [illustrated in this figure].
Abbreviations: PCAR, reporting-standard-adjusted psychiatric care affiliation rates; OutInQuota, the proportion of reporting-standard-adjusted psychiatric visits to outpatient and inpatient facilities.

© 2023 American Medical Association. All rights reserved.

eFigure 8. Diagnostic Assessment of Post Hoc Bipolar-Lithium Model (Including the Regional Yearly Number of Unique Patients Receiving at Least 1 Lithium Prescription as a Covariate): DHARMa Test for Dispersion, QQ-Plots Residuals, and Residuals vs Predicted Lines (Males)

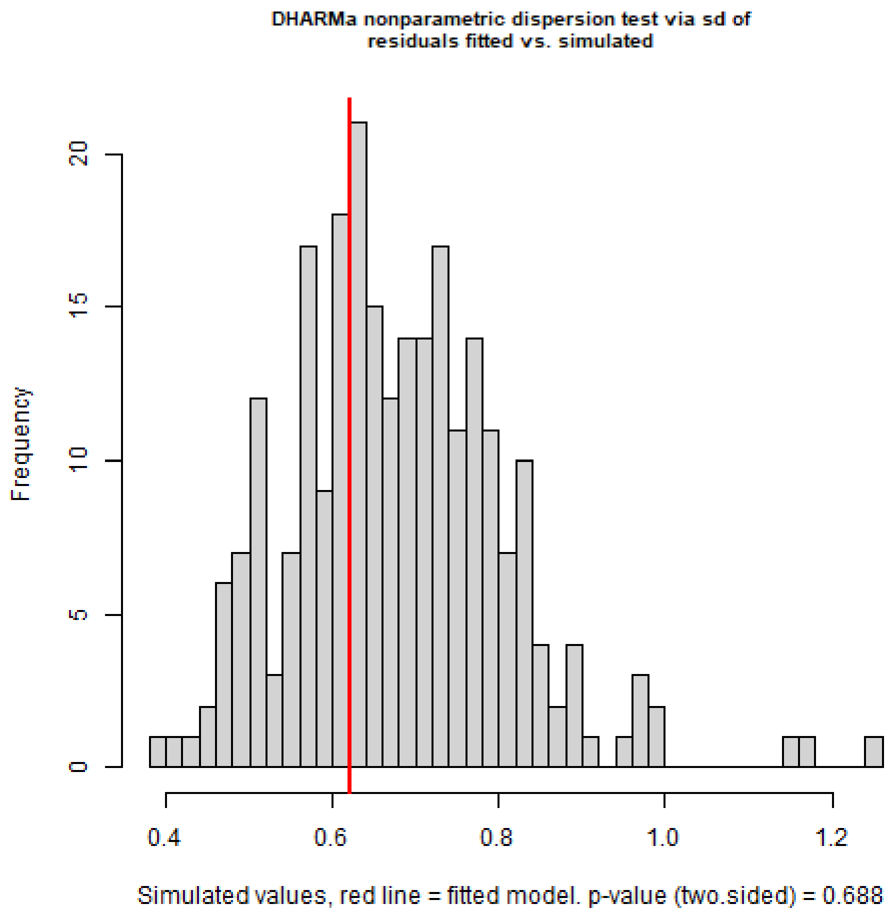

© 2023 American Medical Association. All rights reserved.

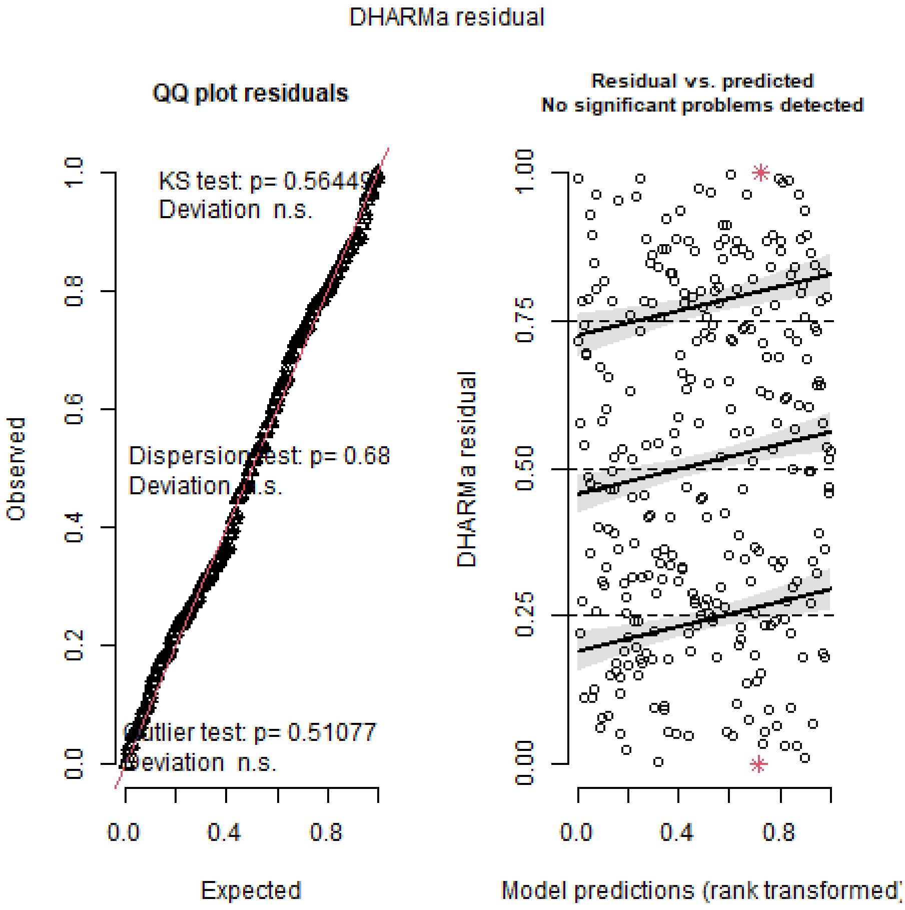

Legend: Associations between the outcome variable (bipolar diagnosis rates), exposures (an interaction term between lithium dispensation frequencies [Lithium treatment rate] and the number of patients receiving lithium treatment at least once in a year [#Lithium treated patients], and an interaction term between PCAR and OutInQuota) were investigated in males by generalized linear mixed effects models, whereby exposure variable were designated as independent fixed-effects variables. Random-intercept effect modifiers pertained to region and year. The model was specified as zero-inflated on the #Lithium treated patients variable, due to a substantial number of zeroes. To avoid violating model assumptions, normalization was applied using Blom's method[17] on the following variables: Lithium treatment rate, PCAR and OutInQuota. This model was tested post-hoc for dispersion and heteroscedasticity – demonstrating no significant problems [illustrated in this figure].
Abbreviations: PCAR, reporting-standard-adjusted psychiatric care affiliation rates; OutInQuota, the proportion of reporting-standard-adjusted psychiatric visits to outpatient and inpatient facilities.

© 2023 American Medical Association. All rights reserved.

eTable 1. Associations Between Lithium Utilization Rates and Bipolar Disorder Diagnosis Frequencies

		Coef.	Std. Error	z value	P-value
Males	(Intercept)	4.146	0.115	35.950	< 2e-16
	**Lithium treatment rate**	**-0.001**	**0.000**	**-2.740**	**0.006**
	PCAR*	0.076	0.066	1.160	0.245
	OutInQuota*	0.027	0.064	0.420	0.674
	PCAR*:OutInQuota*	-0.040	0.034	-1.150	0.250

Dispersion parameter for tweedie family (): 13.7
AIC: N/A
BIC: N/A
logLik: N/A
deviance: N/A
df.resid: N/A

Random effects:
Conditional Model:

**Groups**	**Name**	**Variance**	**Std.Dev**
Year:Weights:Region	(Intercept)	7.46E-09	8.64E-05
Weights:Region	(Intercept)	0.063	0.252
Weights	(Intercept)	0.063	0.252
Region	(Intercept)	0.063	0.252

Number of obs: 294, groups: Year:Weights:Region, 294; Weights:Region, 21; Weights, 21; Region, 21

		Coef.	Std. Error	z value	P-value
Females	(Intercept)	5.080	0.104	48.820	<2e-16
	Lithium treatment rate	0.000	0.000	0.400	0.691
	**PCAR***	**0.120**	**0.049**	**2.450**	**0.015**
	OutInQuota*	0.038	0.051	0.760	0.449
	PCAR*:OutInQuota*	-0.018	0.028	-0.640	0.522

Dispersion parameter for tweedie family (): 16.1
AIC: 3349.3
BIC: 3382.4
logLik: -1665.6
deviance: 3331.3
df.resid: 285

© 2023 American Medical Association. All rights reserved.

Random effects:

Conditional Model:

**Groups**	**Name**	**Variance**	**Std.Dev**
Year:Weights:Region	(Intercept)	7.46E-09	8.64E-05
Weights:Region	(Intercept)	0.063	0.252
Weights	(Intercept)	0.063	0.252
Region	(Intercept)	0.063	0.252
Number of obs: 294, groups: Year:Weights:Region, 294; Weights:Region, 21; Weights, 21; Region, 21			

Associations between the outcome variable (bipolar diagnosis rates), exposures (lithium dispensation frequencies and an interaction term between PCAR and OutInQuota) were investigated in females by generalized linear mixed effects models, whereby exposure variable were designated as independent fixed-effects variables. Random-intercept effect modifiers pertained to region and year. In this analysis, the following variables were subjected to normalization by Blom's method(26) to avoid violating model assumptions: Lithium dispensation rates, PCAR and OutInQuota. This model was tested post-hoc for dispersion, heteroscedasticity, and zero-inflation, evincing no such signs (**Supplemental Figures 4-5.**). *Abbreviations*: Coef., coefficient; Std. Error, standard error; PCAR, reporting-standard-adjusted psychiatric care affiliation rates; N/A, not available; OutInQuota, the proportion of reporting-standard-adjusted psychiatric visits to outpatient and inpatient facilities.

© 2023 American Medical Association. All rights reserved.

eTable 2. Post Hoc Analysis: Associations between Lithium Utilization Rates, Number of Lithium-Treated Patients, and Bipolar Disorder Diagnosis Frequencies (Males)

		Coef.	Std. Error	z value	P-value
Males	(Intercept)	3.851	0.078	49.340	< 2e-16
	**#Lithium treated patients**	**0.008**	**0.001**	**6.980**	**0.000**
	**Lithium treatment rate**	**-0.162**	**0.050**	**-3.240**	**0.001**
	PCAR*	0.024	0.053	0.450	0.653
	OutInQuota*	0.017	0.050	0.340	0.730
	Lithium (patients):Lithium (treatment rate)*	0.001	0.001	0.750	0.451
	PCAR*:OutInQuota*	-0.024	0.027	-0.870	0.386
	Dispersion parameter for tweedie family (): 7.08				
	**Random effects:**				
	Conditional Model:				
	**Groups**	**Name**	**Variance**	**Std.Dev**	
	Year:Region	(Intercept)	0.033	0.181	
	Region	(Intercept)	0.076	0.028	
	Number of obs: 294, groups: Year:Weights:Region, 294; Weights:Region, 21; Weights, 21; Region, 21				

Associations between the outcome variable (bipolar diagnosis rates), exposures (an interaction term between lithium dispensation frequencies [Lithium treatment rate] and the number of patients receiving lithium treatment at least once in a year [#Lithium treated patients], and an interaction term between PCAR and OutInQuota) were investigated in males by generalized linear mixed effects models, whereby exposure variable were designated as independent fixed-effects variables. Random-intercept effect modifiers pertained to region and year. Fixed effects variables that were subjected to transformation by Blom's methods to allow violation of model assumptions are highlighted by asterix (*) This model was tested post-hoc for dispersion, heteroscedasticity, and zero-inflation, evincing no such signs (**Supplemental Figures 8.**).
Abbreviations: Coef., coefficient; Std. Error, standard error; PCAR, reporting-standard-adjusted psychiatric care affiliation rates; OutInQuota, the proportion of reporting-standard-adjusted psychiatric visits to outpatient and inpatient facilities.

© 2023 American Medical Association. All rights reserved.

eTable 3. Post Hoc Analysis: Associations Between Adolescent Suicide Mortality, Bipolar Disorder and Major Depressive Disorder (MDD)/Schizophrenia Diagnosis Frequencies, and Lithium Utilization Rates (Males)

		Coef.	Std. Error	z value	P-value
Males	(Intercept)	2.544	0.146	17.454	<2e-16
	**Bipolar Diagnosis Rate**	**-0.004**	**0.002**	**-2.180**	**0.0293**
	Lithium Treatment Rate*	-0.207	0.122	-1.699	0.0893
	MDD/Schizophrenia Diagnosis Rate*	0.001	0.101	0.013	0.9897
	PCAR*	0.006	0.124	0.049	0.9608
	OutInQuota*	-0.011	0.100	-0.110	0.9121
	Bipolar Diagnosis Rate:Lithium Treatment Rate*	0.001	0.002	0.730	0.4655
	PCAR*:OutInQuota*	-0.062	0.067	-0.923	0.3559

Dispersion parameter for tweedie family (): 8.66
AIC: 1713.1
BIC: 1757.3
logLik: -844.5
deviance: 1689.1
df.resid: 282

Random effects:
Conditional Model:

**Groups**	**Name**	**Variance**	**Std.Dev**
Year:Region	(Intercept)	0.147	0.384
Region	(Intercept)	1.636E-08	0.000

Number of obs: 294, groups: Year:Region, 294; Region, 21

The association analyses between adolescent suicide mortality rates and BD diagnosis frequencies and suicide death rates were performed separately in females and males, applying generalized linear mixed effects models modeled on the Tweedie-distribution whereby the yearly regional BD diagnosis frequencies, the yearly regional number of lithium dispensations, and the combined yearly regional number of MDD (ICD-10: F32) and schizophrenia (ICD-10: F20) diagnosis frequencies were designated as fixed-effects variables (interaction term between the former two variables). An independent interaction term between PCAR and OutInQuota were further included as fixed-effects variables. Region and year were included as random-intercept effects. Fixed effects variables were subjected to transformation by Blom's methods and are highlighted by asterix (*). The model did not evince any signs of bias from overdispersion or heteroscedasticity – and the zero-inflation assumption was confirmed as valid (**Supplemental Figure 6.**).
Abbreviations: Coef., coefficient; MDD, major depressive disorder; Std. Error, standard error; PCAR, reporting-standard-adjusted psychiatric care affiliation rates; OutInQuota, the proportion of reporting-standard-adjusted psychiatric visits to outpatient and inpatient facilities.

© 2023 American Medical Association. All rights reserved.

eTable 4. Post Hoc Analysis: Associations in Males Between Adolescent Suicide Mortality, Bipolar Disorder and Major Depressive Disorder (MDD)/Schizophrenia Diagnosis Frequencies, and Lithium Utilization Rates, a Generalized Linear Mixed-Effects Model Modeled on the β-Binomial Distribution

		Coef.	Std. Error	z value	P-value
Males	(Intercept)	-0.963	0.169	-5.702	0.000
	**Bipolar Diagnosis Rate***	**-0.466**	**0.161**	**-2.902**	**0.004**
	Lithium Treatment Rate*	-0.064	0.158	-0.405	0.686
	MDD/Schizophrenia Diagnosis Rate*	0.156	0.220	0.709	0.479
	PCAR*	-0.192	0.277	-0.691	0.490
	OutInQuota*	0.081	0.206	0.392	0.695
	Bipolar Diagnosis Rate:Lithium Treatment Rate*	0.126	0.169	0.745	0.456
	PCAR*:OutInQuota*	-0.265	0.151	-1.749	0.080
	Dispersion parameter for betabinomial (): 1				
	**Random effects:**				
	Conditional Model:				
	**Groups**	**Name**	**Variance**	**Std.Dev**	
	Year:Region	(Intercept)	5.98E-08	2.45E-04	
	Region	(Intercept)	0.019	0.139	
	Number of obs: 294, groups: Year:Region, 294; Region, 21				

ASM was dichotomized based on the 75th percentile – whereby top-quartile (Q4) observations regarding ASM were compared to lower-quartile (Q1-Q3) observations.The association analyses between dichotomized adolescent suicide mortality rates and BD diagnosis frequencies were investigated in males, applying generalized linear mixed effects models modeled on the Tweedie-distribution whereby the yearly regional BD diagnosis frequencies, the yearly regional number of lithium dispensations and the combined yearly regional number of MDD (ICD-10: F32) and schizophrenia (ICD-10: F20) diagnosis frequencies were designated as a fixed-effects variables (interaction term between the two former variables). An independent interaction term between PCAR and OutInQuota were further included as fixed-effects variables. Region and year were included as random-intercept effects. Fixed effects variables were subjected to transformation by Blom's methods and are highlighted by asterix (*). The model did not evince any signs of bias from overdispersion or heteroscedasticity – and the zero-inflation assumption was confirmed as valid (**Supplemental Figure 7.**).
Abbreviations: Coef., coefficient; MDD, major depressive disorder; Std. Error, standard error; PCAR, reporting-standard-adjusted psychiatric care affiliation rates; OutInQuota, the proportion of reporting-standard-adjusted psychiatric visits to outpatient and inpatient facilities.

© 2023 American Medical Association. All rights reserved.
