# Supplementary material for: Association of Bipolar Disorder Diagnosis With Suicide Mortality Rates in Adolescents in Sweden
Source: JAMA Psychiatry. 2023 May 24;80(8):796–802. doi: 10.1001/jamapsychiatry.2023.1390 (PMC10209824; doi:10.1001/jamapsychiatry.2023.1390)
Supplement: Supplement 2. — Data Sharing Statement [file jamapsychiatry-e231390-s002.pdf]

Data Sharing Statement

Andersson. Association of Reduced Regional Suicide Mortality With Bipolar Disorder Diagnosis Rates in Adolescents in Sweden. *JAMA Psychiatry*. Published May 24, 2023. doi:10.1001/jamapsychiatry.2023.1390

Data
Data available: Yes
Data types: Deidentified participant data
How to access data: To obtain access to the deidentified participant data used in this study, interested researchers can submit a request to the data custodian at adrian.desai.bostrom@ki.se. Upon approval of the request, the data will be made available in accordance with the applicable ethical and legal requirements.
When available: With publication

Supporting Documents
Document types: Statistical/analytic code
How to access documents: To obtain access to the analysis code used in this study, interested researchers can request it from the corresponding author at adrian.desai.bostrom@ki.se. Upon approval of the request, the code will be made available in accordance with the applicable ethical and legal requirements.
When available: With publication

Additional Information
Who can access the data: Anyone requesting the data
Types of analyses: For any purpose.
Mechanisms of data availability: After approval of a proposal.
Any additional restrictions: None.
